# Finite-Horizon Reliability-Oriented Synthesis of Cumulative Up/Down-Counter Fault-Confirmation Monitors

**DOI:** 10.3390/s26165115

**Published:** 2026-08-12

**Authors:** Xiaoting Yuan, Xiaotong Feng, Ming Cheng, Peng Wang

**Affiliations:** 1 Shanghai Aircraft Design and Research Institute, Shanghai 201210, China; yuanxiaoting@comac.cc; 2 Department of Integrated Circuits Science and Engineering, Beihang University, Beijing 100191, China; betty513@buaa.edu.cn; 3 Department of Environment, Chemistry and Materials, University of Bologna, 40130 Bologna, Italy; ming.cheng3@unibo.it

**Keywords:** LVDT, fault-confirmation monitor, counter-based alarm mechanism, finite-horizon reliability, kernel density estimation, Markov chain

## Abstract

Up/down counters are ubiquitous in the alarm and fault-confirmation logic of electro-mechanical systems. In aircraft, several electro-mechanical modules provide position feedback for flight control; the Linear Variable Differential Transformer (LVDT) is a representative one, converting mechanical displacement into an electrical signal whose reliable monitoring is critical to flight safety. As such counters are deployed in ever more complex systems and more uncertain environments, rising safety requirements render their heuristic tuning unreliable. To address this challenge, this paper proposes a quantitative, reliability-oriented procedure for counter-based monitors, which replaces heuristic parameter tuning. Both healthy and faulty signal distributions are estimated by Kernel Density Estimation (KDE), so the framework handles non-Gaussian noise and FMEA-weighted failure modes. The threshold-and-counter logic is modeled as a finite-horizon absorbing Discrete-Time Markov Chain (DTMC), which yields the false-confirmation probability, missed-detection probability, and detection delay over a bounded horizon instead of long-run rates. Thresholds and counter parameters are then synthesized offline, leaving a lightweight online monitor that needs only threshold comparison and integer counter updates. We evaluate the method on an LVDT sum-voltage monitor using real aircraft healthy measurements and Simulink-based fault injection with 45 detectable modes, assessing the synthesized monitor on 558,001 real measured samples and an FMEA-driven fault population. Results show that, among the compared confirmation logics, our workflow yields a counter that meets the $10^{-9}$ false-confirmation target and the $10^{-6}$ missed-detection target while attaining the lowest detection delay.

## 1. Introduction

Alarm and fault-confirmation monitors are widely used in industrial and safety-critical systems, ranging from nuclear plants to aerospace flight-control systems. They detect faults and abnormal operating conditions and provide notifications related to system safety and operational status. Industrial alarm analysis and alarm-management guidelines emphasize that missed alarms and alarm delays must be handled jointly during alarm configuration and rationalization [1,2,3]. Recent work also provides a systematic treatment of advanced analysis and design methods for intelligent industrial alarm systems [4]. A common lightweight confirmation mechanism is counter-based logic, in which threshold violations are accumulated before an alarm or fault is confirmed.

The performance of such counter- and delay-timer-based mechanisms has been analyzed extensively, yet almost always through steady-state performance evaluation or conventional alarm-filter design rather than parameter synthesis for a finite-horizon requirement—the setting of the present work. Markov-chain models characterize the false-alarm rate (FAR), missed-alarm rate (MAR), and detection delay of a fixed configuration. Early studies established these indices and the Markov-chain machinery for deadband and delay-timer alarms [5,6,7]. Later studies refined the delay-timer and deadband designs and their risk trade-offs [8,9,10,11,12]. More recently, Zhou et al. [13] derived expressions for the false-alarm and fault-detection rates of stochastic anomaly detectors with on/off-delay timers for temporally correlated process variables. Most relevant here, Raei et al. [14] examined up/down-counter configurations for univariate alarm systems, while Rahimi et al. [15] compared the performance of delay timers, counters, and time deadbands. The analytical toolbox has also been extended to cases in which the memoryless assumption fails, with variable-threshold systems evaluated through semi-Markov processes [16] and joint threshold and delay-timer design addressed for non-IID process variables based on alarm-duration statistics [17]. Asaadi et al. [18] also assessed alarm systems for mixture processes and intermittent faults using time-varying finite-mixture models. Collectively, these studies address the evaluation, design, and comparison of individual alarm-filter structures across increasingly complex process conditions.

Despite this breadth, two gaps remain for the setting considered here. First, prior alarm studies generally focus on recurrent alarm operation, long-run performance assessment, or conventional alarm-filter design. By contrast, embedded fault confirmation is a latched trip within a bounded horizon, for which the relevant risk is the first confirmation event. This finite-horizon, first-passage viewpoint is central to classical sequential change detection [19,20,21,22]. Markov-chain embedding for run-length and first-passage events [23] provides the computational basis. However, the alarm literature has not used these tools to synthesize up/down-counter parameters under finite-horizon reliability constraints. Second, counter-based confirmation is well established in electro-mechanical fault diagnosis. Ozdemir et al. [24] showed that it detects smaller faults at a lower false-alarm rate than fixed thresholds. Nevertheless, its counting steps and trip limit are still set by hand. No end-to-end procedure currently maps signal distributions and fault statistics to a reliability-constrained choice of the thresholds and counter parameters (X,Y,H).

These gaps are sharpened in modern electro-mechanical systems, whose many sensors, actuators, and signal-conditioning components make purely heuristic tuning inadequate. A representative case is the actuation channel of aircraft flight-control systems. In this channel, a Linear Variable Differential Transformer (LVDT) converts the mechanical position of the control surface into an electrical signal that must be conditioned and demodulated [25,26]. A fault in its sensing or conditioning circuit can corrupt the position feedback and, in turn, the attitude control of the aircraft. Reliable fault detection and isolation in such channels is a long-standing aerospace concern [27,28]. A practical monitor must simultaneously handle non-Gaussian signal variability, FMEA-guided fault populations, and a stringent false-confirmation requirement. A recent review of prognostics and health management for critical aircraft systems and components likewise emphasizes proactive condition monitoring and fault prognosis [29]. Complementary work on safety-critical electro-mechanical systems has studied fault-tolerant control, attack resilience, and state and fault estimation after faults or attacks affect the plant [30,31,32]. The proposed amplitude-and-counter monitor provides the first fault-confirmation step and complements these higher-level methods. A deployable design procedure must therefore meet these requirements jointly while keeping the online logic as lightweight as the original threshold-and-counter mechanism.

To address these challenges, this paper proposes a reliability-oriented parameter synthesis framework for threshold-and-counter LVDT fault-confirmation monitoring. The framework combines engineering-informed monitored-signal distribution modeling, finite-horizon DTMC reliability evaluation, and two-stage offline parameter synthesis. The main contributions are summarized as follows:

1. End-to-end reliability-oriented workflow: We combine FMEA-guided distribution modeling, finite-horizon reliability evaluation, and offline synthesis into one workflow, replacing heuristic counter tuning with a quantitative configuration.
2. Finite-horizon DTMC reliability evaluation: The threshold-and-counter logic is modeled as an absorbing DTMC, giving finite-horizon false-confirmation, missed-detection, and detection-delay metrics instead of classical long-run rates.
3. Two-stage offline synthesis with lightweight deployment: Threshold selection and counter-parameter optimization are done offline under reliability constraints, leaving an online monitor that needs only threshold comparisons and integer counter updates.

The rest of this paper is organized as follows. Section 2 formulates the problem and the finite-horizon reliability metrics, Section 3 presents the two-stage synthesis method, Section 4 reports the LVDT case study, Section 5 discusses the application methodology, performance trade-offs, sensor-level errors, and study limitations, and Section 6 concludes.

## 2. Problem Formulation and Preliminaries

### 2.1. Counter-Based Confirmation Mechanisms

The monitor considered in this work belongs to a broader class of counter-based confirmation mechanisms. By choosing different recovery rules, the same counter update equation can describe either cumulative up/down counting or consecutive-sample triggering. In this paper, the cumulative up/down counter is the primary design object, whereas the consecutive rule is included only to show its connection with delay-timer-type baselines. The objective of this work is not to replace this lightweight engineering monitor with a complex online diagnosis algorithm, but to quantitatively synthesize its amplitude thresholds and confirmation parameters so that the finite-horizon false-confirmation and missed-detection probabilities can be evaluated and controlled within a bounded monitoring horizon.

Mathematically, let $s_{k}\in R$ denote the monitored scalar signal at sampling instant *k*. For the LVDT sum-voltage monitor used as the validation scenario in this work, $s_{k}$ can be written as

(1) $$s_{k}=V_{a,k}+V_{b,k},$$

where $V_{a,k}$ and $V_{b,k}$ are the two monitored voltage components. In this work, the monitor directly evaluates the sum-voltage signal $s_{k}$ against prescribed lower and upper amplitude thresholds.

Given a lower amplitude threshold *L* and an upper amplitude threshold *U*, with L<U, the threshold-violation indicator is defined as

(2) $$\delta_{k}=\delta \left(s_{k};L,U\right)=\left\{\begin{array}{cc} 1, & s_{k}\notin \left[L,U\right],\\ 0, & s_{k}\in \left[L,U\right]. \end{array}\right.,$$

here, $\delta_{k}=1$ indicates that the current signal sample $s_{k}$ violates the admissible range, whereas $\delta_{k}=0$ indicates that it remains within the admissible range.

Let $C_{k}\in \left\{0,1,\ldots ,H\right\}$ denote the counter state used by the fault-confirmation logic, where *H* is the trip limit. Let *X* denote the upward counting step under a threshold violation, and let Rec(·) denote the recovery rule when no violation occurs. The counter update rule can be written as

(3) $$C_{k+1}=\left\{\begin{array}{cc} \min(H,C_{k}+X), & \delta_{k}=1,\\ \mathrm{Rec}(C_{k}), & \delta_{k}=0, \end{array}\right.$$

with initial condition $C_{0}=0$.

The recovery rule determines the specific confirmation mechanism:

(4) $$\mathrm{Rec}\left(C_{k}\right)=\left\{\begin{array}{cc} \max(0,C_{k}-Y), & \mathrm{cumulative}\;\mathrm{up}/\mathrm{down}\;\mathrm{counting},\\ 0, & \mathrm{consecutive}\text{-}\mathrm{sample}\;\mathrm{triggering}, \end{array}\right.$$

where Y>0 is the down step. In cumulative up/down counting, a normal sample decreases the counter by *Y* when no violation happens. Consecutive-sample triggering instead resets the counter to zero on any normal sample, corresponding to delay-timer-type logic.

A fault is confirmed once the counter reaches the trip limit *H*, and the first fault-confirmation time is

(5) $$\tau_{H}=\inf\left\{k\geq 0:C_{k}=H\right\}.$$

The confirmed state is treated as absorbing within the monitoring window, since the objective is whether a fault is confirmed within the prescribed horizon, not how the alarm is cleared afterward, consistent with the latching semantics of embedded fault-confirmation monitors.

$p_{i}=p_{i}\left(L,U\right)$ is the single-sample threshold-violation probability defined in Equations (7) and (8). The absorbing-DTMC transition matrix $P_{i}\left(\Theta \right)\in R^{\left(H+1)\times (H+1\right)}$ is built from $p_{i}\left(L,U\right)$ (with $p_{0}$ for $H_{0}$ and $p_{1}$ for $H_{1}$), where $H_{0}$ and $H_{1}$ denote the healthy and faulty operating conditions. For c,j∈{0,1,…,H},

(6) $${\left[P_{i}\left(\Theta \right)\right]}_{c,j}=\left\{\begin{array}{cc} p_{i}, & j=\min(H,c+X),\;c=0,\ldots ,H-1,\\ 1-p_{i}, & j=\mathrm{Rec}(c),\;c=0,\ldots ,H-1,\\ 1, & c=H,\;j=H,\\ 0, & \mathrm{otherwise}. \end{array}\right..$$

Before absorption, a violation moves the counter up with probability $p_{i}$ and a non-violation applies the recovery rule with probability $1-p_{i}$; state *H* is absorbing. Coinciding target states are merged.

For the cumulative counter, the transition matrix is still defined on the full state space {0,1,…,H} by Equation (6), where Θ=(L,U,X,Y,H). To reduce the offline propagation cost, we first identify the states reachable from the initial state $C_{0}=0$. Starting from $S_{0}=\left\{0\right\}$, the reachable set is generated by

$$S_{n+1}=S_{n}\cup \underset{c\in S_{n},\;c<H}{⋃}\left\{\min(H,c+X),\;\max(0,c-Y)\right\},$$

and the reachable-state set is

$$S_{\mathrm{reach}}=\underset{n\geq 0}{⋃}S_{n},$$

while the full (H+1)×(H+1) matrix indexing is retained. Before state propagation, all matrix entries associated with unreachable states are masked and set to zero, while the entries corresponding to $S_{\mathrm{reach}}$ are left unchanged. Since no probability mass can enter an unreachable state from the initial condition $C_{0}=0$, this zeroing operation does not change the finite-horizon values of $P_{FA}$, $P_{MD}$, or ADD. It only reduces the number of non-zero entries used during offline propagation. The online monitor remains unchanged.

### 2.2. Single-Sample Violation Probabilities

Given $H_{0}$ and $H_{1}$ operating conditions, respectively, the single-sample threshold-violation probabilities under $H_{0}$ and $H_{1}$ are defined as

(7) $$p_{0}\left(L,U\right)=P\left(\delta_{k}=1|H_{0}\right)=P\left(s_{k}\notin \left[L,U\right]|H_{0}\right),$$

and

(8) $$p_{1}\left(L,U\right)=P\left(\delta_{k}=1|H_{1}\right)=P\left(s_{k}\notin \left[L,U\right]|H_{1}\right).$$

If the signal probability density functions under $H_{0}$ and $H_{1}$ are denoted by $f_{0}\left(s\right)$ and $f_{1}\left(s\right)$, respectively, then

(9) $$p_{0}\left(L,U\right)=1-\int_{L}^{U}f_{0}\left(s\right)\;ds,$$

and

(10) $$p_{1}\left(L,U\right)=1-\int_{L}^{U}f_{1}\left(s\right)\;ds.$$

I.e., the single-sample violation probability is the probability mass outside the admissible threshold interval.

This formulation does not require $f_{0}$ and $f_{1}$ to be Gaussian. In this work, these distributions can be obtained from engineering-informed probabilistic approximations, where nominal data, input uncertainty, and in-process noise are embedded into the probabilistic description of healthy and faulty signal behavior. In particular, $f_{1}\left(s\right)$ can represent a mixed or aggregated fault distribution that integrates multiple fault modes and their variability.

For tractability, the threshold-violation sequence is first modeled as an independent Bernoulli process under each hypothesis:

(11) $$\delta_{k}|H_{i}∼\mathrm{Bernoulli}\;\left(p_{i}\left(L,U\right)\right),\;i\in \left\{0,1\right\}.$$

Consequently, the continuous signal distribution affects the counter only through the scalar violation probability $p_{i}\left(L,U\right)$.

Therefore, the amplitude thresholds and uncertainty characterization are coupled through $p_{0}\left(L,U\right)$ and $p_{1}\left(L,U\right)$: a conservative band lowers $p_{0}$ and suppresses nuisance trips but weakens sensitivity to weak or slow faults, whereas an aggressive band improves sensitivity at the cost of a higher false-confirmation risk. These threshold-induced probabilities serve as the probabilistic inputs to the DTMC reliability model.

### 2.3. Finite-Horizon Reliability Metrics

A false confirmation occurs when the monitor trips under the healthy condition $H_{0}$, and a missed detection occurs when it fails to trip under the faulty condition $H_{1}$. Because the present monitor confirms and latches a fault within a bounded window, we define finite-horizon probabilities rather than long-run rates.

Let $T_{h}$ denote the prescribed monitoring horizon; the key event is whether the counter reaches the trip limit within $T_{h}$ samples. Under $H_{0}$, this corresponds to a false confirmation, and the finite-horizon false-confirmation probability is

(12) $$P_{FA}\left(T_{h};\Theta \right)=P\left(\tau_{H}\leq T_{h}|H_{0}\right),$$

where Θ is the monitor parameter set. $P_{FA}\left(T_{h};\Theta \right)$ is a finite-window probability, not a long-run rate.

Under the faulty condition $H_{1}$, reaching the trip limit within $T_{h}$ samples corresponds to successful fault confirmation, so the finite-horizon missed-detection probability is

(13) $$P_{MD}\left(T_{h};\Theta \right)=P\left(\tau_{H}>T_{h}|H_{1}\right)=1-P\left(\tau_{H}\leq T_{h}|H_{1}\right).$$

In addition, the average detection delay under the faulty condition is defined as

(14) $$\mathrm{ADD}\left(\Theta \right)=E\left[\tau_{H}|H_{1},\;\tau_{H}\leq T_{h}\right].$$

I.e., the expected number of samples to confirmation, conditioned on detection within the horizon. Missed cases ($\tau_{H}>T_{h}$) are captured by $P_{MD}\left(T_{h};\Theta \right)$ and excluded from ADD, so ADD is meaningful only together with $P_{MD}$; configurations violating the missed-detection constraint should not be ranked by ADD alone. This conditional definition is used throughout the experiments.

Let $e_{0}$ and $e_{H}$ be the canonical unit vectors for the initial state 0 and the absorbing trip state *H*. Since *H* is absorbing, the finite-horizon probabilities are

(15) $$P_{FA}\left(T_{h};\Theta \right)=e_{0}^{⊤}P_{0}{\left(\Theta \right)}^{T_{h}}e_{H},$$

and

(16) $$P_{MD}\left(T_{h};\Theta \right)=1-e_{0}^{⊤}P_{1}{\left(\Theta \right)}^{T_{h}}e_{H}.$$

Unlike classical alarm analysis based on long-run rates for recurrent alarm systems, the trip state here is a latched event within a finite horizon, so the relevant risk is the first-hitting probability within $T_{h}$ rather than the steady-state alarm probability.

### 2.4. Monitor Parameter Set and Feasible Domain

For the cumulative up/down counter emphasized in this work, the parameter set is

(17) Θ=(L,U,X,Y,H),

where L,U are the amplitude thresholds, X,Y the upward and downward counting steps, and *H* the trip limit. Throughout, a superscript star (e.g. $L^{★},U^{★},X^{★},Y^{★},H^{★}$) denotes a quantity synthesized by the offline procedure, whereas quantities specified by the designer as fixed inputs—the reliability targets α,β, the monitoring horizon $T_{h}$, the guard factor $k_{g}$, and the search ranges X,Y,H. This distinction separates the design choices a user must supply from the parameters the method returns. A typical feasible domain is

(18) $$\Omega_{\mathrm{cum}}=\left\{\left(L,U,X,Y,H\right):\begin{array}{c} L\in L,\;U\in U,\;L<U,\\ X,Y,H\in Z_{>0},\\ X\leq H,\;Y\leq H,\;H\leq H_{\max} \end{array}\right\},$$

where L,U are the allowable engineering ranges and $H_{\max}$ is the maximum implementable counter value.

In summary, this section has reduced the threshold-and-counter monitor to the single-sample violation probabilities $p_{0}\left(L,U\right)$ and $p_{1}\left(L,U\right)$, which parameterize the absorbing DTMC and yield the finite-horizon metrics $P_{FA}$, $P_{MD}$, and ADD for any candidate configuration $\Theta \in \Omega_{\mathrm{cum}}$. How Θ is synthesized from these metrics is the subject of the next section.

## 3. Reliability-Oriented Parameter Synthesis Method

**Figure 1:**
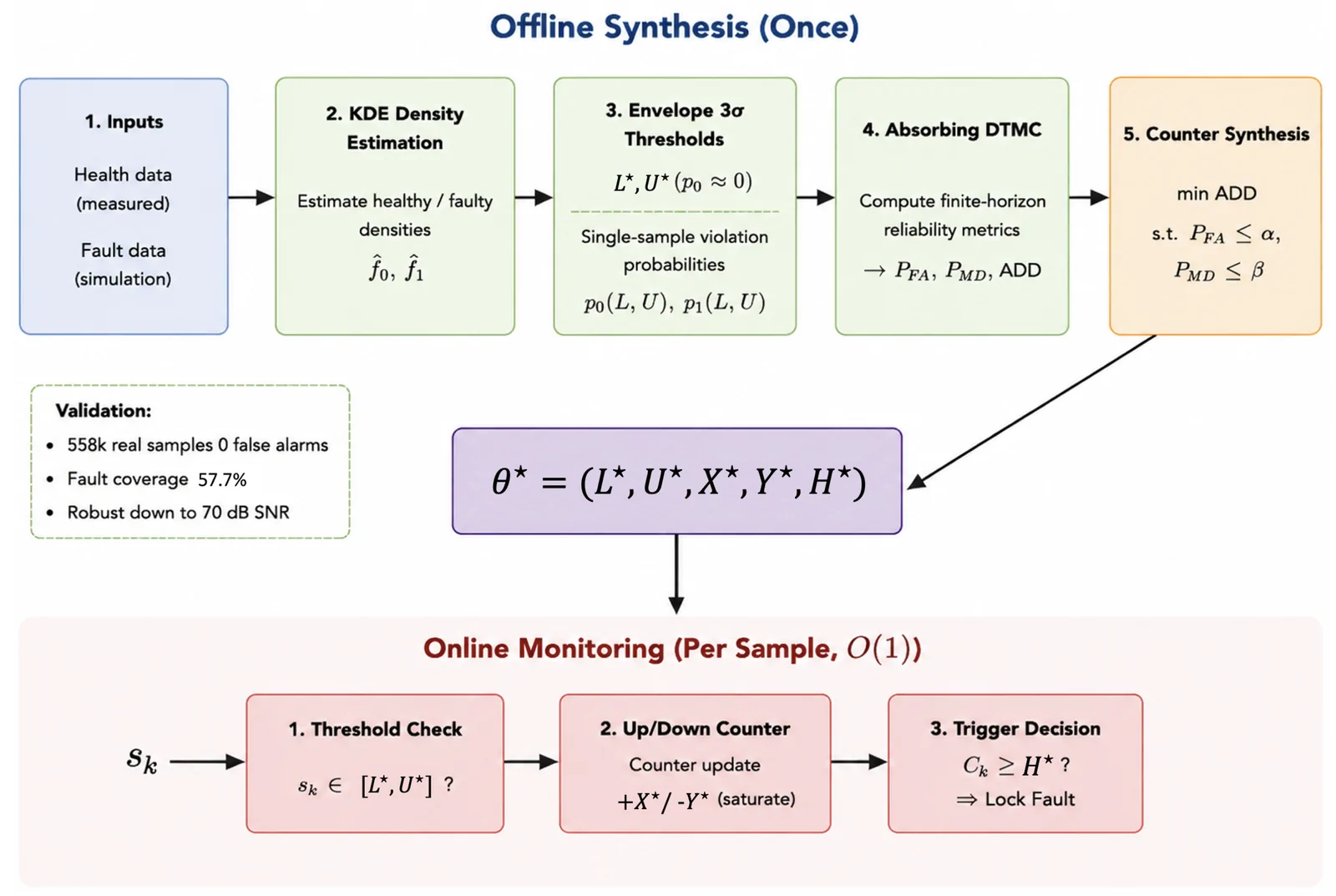
Overview of the proposed reliability-oriented synthesis framework.

The previous section formulated the threshold-and-counter monitor as a finite-horizon reliability synthesis problem. This section presents the proposed end-to-end synthesis workflow that solves it. Figure 1 gives an overview: measured healthy data and simulated faulty data are first turned into signal densities, then into single-sample violation probabilities, which drive the absorbing-DTMC reliability evaluation and the two-stage offline parameter synthesis; the deployed online monitor retains only the lightweight threshold-and-counter logic.

Supporting analyses are organized in their citation order. Per-mode fault coverage and detectability are reported in Appendix A; the FMEA, threshold-selection, subsampling, and healthy-data validation results are provided in Appendix B; and the numerical DTMC verification and search-efficiency results are reported in Appendix C.

### 3.1. Novel Reliability-Oriented Workflow

The proposed method integrates FMEA-guided signal-distribution modeling, absorbing-DTMC reliability evaluation, and two-stage parameter synthesis into a single design procedure with an offline–online structure. In the offline phase, the healthy density ${\hat{f}}_{0}\left(s\right)$ is mapped to $p_{0}\left(L,U\right)$, whereas each mode-specific faulty density ${\hat{f}}_{1,\ell }\left(s\right)$ is mapped to its own violation probability $p_{1,\ell }\left(L,U\right)$ for mode-wise reliability evaluation. The FMEA-weighted density ${\hat{f}}_{1}\left(s\right)$ is used only for population-level distribution and occurrence-weighted coverage analysis. The amplitude thresholds $\left(L^{★},U^{★}\right)$ are then selected to contain the healthy operating range with a guard band, after which the counter parameters $\left(X^{★},Y^{★},H^{★}\right)$ are synthesized by minimizing the detection delay subject to the finite-horizon false-confirmation and mode-wise missed-detection constraints. In the online phase, the deployed monitor preserves the original lightweight structure, requiring only threshold comparison, a saturated integer counter update, and trip checking. The five offline steps of Figure 1 are detailed in Section 3.2, and the online deployment is summarized in Section 3.3.

### 3.2. Offline Synthesis

#### 3.2.1. Signal-Distribution Modeling from Measured and Simulated Data

This first workflow stage (Steps 1–2 in Figure 1) turns the input data, measured healthy signals and simulated faulty signals, into the healthy and faulty densities ${\hat{f}}_{0}\left(s\right)$ and ${\hat{f}}_{1}\left(s\right)$ by Kernel Density Estimation (KDE). Instead of assuming a fixed Gaussian form, engineering information, nominal data, input uncertainty, process noise, and representative fault modes are used to construct the distributions required for reliability evaluation.

##### Data Preparation and Controlled Noise Modeling

When measured healthy data and clean simulation responses are both available, they are synchronized over their common interval and the simulation is interpolated onto the measurement grid. An affine gain–bias calibration then reduces systematic mismatch:

(19) $$s_{k}^{\mathrm{real}}\approx a\;s_{k}^{\mathrm{sim}}+b,$$

where *a* and *b* are estimated by ordinary least squares from the *N* aligned healthy sample pairs:

(20) $$\left(\hat{a},\hat{b}\right)=\arg\min_{a,b}\sum_{k=1}^{N}{\left(s_{k}^{\mathrm{real}}-a\;s_{k}^{\mathrm{sim}}-b\right)}^{2}$$

The coefficients obtained from Equation (20) define the calibration in Equation (19), which maps the clean Simulink output to the scale of the measured healthy output. The affine form is used first because it is the lowest-complexity mapping capable of correcting both gain and offset. If its residual error is not sufficiently small, second- or third-order polynomial extensions may be considered; higher-order terms are retained only when they materially reduce the residual error. The corresponding comparison is reported in Appendix C.1.

The calibrated simulation output is $s_{k}^{\mathrm{cal}}=\hat{a}\;s_{k}^{\mathrm{sim}}+\hat{b}$, and the residual discrepancy is

(21) $${\hat{d}}_{k}=s_{k}^{\mathrm{real}}-s_{k}^{\mathrm{cal}},$$

which represents the aggregate effect of measurement noise, residual model mismatch, and interpolation and alignment errors, rather than pure sensor noise. The corresponding calibration signal-to-discrepancy ratio (SDR) is defined as

(22) $${\hat{\mathrm{SDR}}}_{\mathrm{cal},\mathrm{dB}}=10\log_{10}\;\left(\frac{\frac{1}{N}\sum_{k=1}^{N}{\left(s_{k}^{\mathrm{cal}}\right)}^{2}}{\frac{1}{N}\sum_{k=1}^{N}{\hat{d}}_{k}^{2}}\right).$$

Separately, the fluctuation-based effective SNR of the measured healthy record is used to characterize its observed steady-state signal quality. Let $N_{m}$ denote the number of measured steady-state samples and let ${\bar{s}}^{\mathrm{real}}=N_{m}^{-1}\sum_{k=1}^{N_{m}}s_{k}^{\mathrm{real}}$. This metric is defined as

(23) $${\hat{\mathrm{SNR}}}_{\mathrm{fluc},\mathrm{dB}}=10\log_{10}\;\left(\frac{\frac{1}{N_{m}}\sum_{k=1}^{N_{m}}{\left(s_{k}^{\mathrm{real}}\right)}^{2}}{\frac{1}{N_{m}}\sum_{k=1}^{N_{m}}{\left(s_{k}^{\mathrm{real}}-{\bar{s}}^{\mathrm{real}}\right)}^{2}}\right).$$

The calibration SDR is used to evaluate the accuracy of the simulation-to-measurement mapping, whereas the fluctuation-based SNR characterizes the observed noise and steady-state fluctuation level of the measured healthy data. The latter is used as the nominal measured-data quality level in the robustness experiments.

The same affine mapping is subsequently used to calibrate the fault-injection outputs. Specifically, representative faults are first injected into the Simulink model. After each fault-injection simulation, the resulting faulty Simulink output is transformed according to

$$s_{r,k}^{(1),\mathrm{cal}}=\hat{a}\;s_{r,k}^{(1),\mathrm{sim}}+\hat{b},$$

where $s_{r,k}^{(1),\mathrm{sim}}$ and $s_{r,k}^{(1),\mathrm{cal}}$ denote the original and calibrated outputs of fault-injection case *r*, respectively. The calibrated healthy and faulty outputs are subsequently used for distribution construction, violation probability calculation, and monitor parameter evaluation.

For controlled sensitivity studies, noisy data are generated by adding zero-mean Gaussian noise,

(24) $$s_{k}^{\mathrm{obs}}=s_{k}^{\mathrm{clean}}+n_{k},\;n_{k}∼N\left(0,\sigma_{n}^{2}\right),$$

with the variance for a prescribed test ${\mathrm{SNR}}_{\mathrm{test},\mathrm{dB}}$ set by

(25) $$P_{s}=\frac{1}{N_{s}}\sum_{k=1}^{N_{s}}{\left(s_{k}^{\mathrm{clean}}\right)}^{2},\;\sigma_{n}^{2}=\frac{P_{s}}{10^{{\mathrm{SNR}}_{\mathrm{test},\mathrm{dB}}/10}}.$$

Those steps are used only offline; the deployed monitor performs only threshold comparison and integer counter updates.

##### Healthy Density Estimation

The healthy density $f_{0}\left(s\right)$ captures nominal variability from sensor and process noise, disturbances, and implementation-level fluctuations. Given healthy samples ${\left\{s_{m}^{\left(0\right)}\right\}}_{m=1}^{N_{0}}$ with no parametric form assumed, KDE gives [33,34]

(26) $${\hat{f}}_{0}\left(s\right)=\frac{1}{N_{0}h_{0}}\sum_{m=1}^{N_{0}}K\;\left(\frac{s-s_{m}^{\left(0\right)}}{h_{0}}\right),$$

where K(·) is the kernel and $h_{0}$ the bandwidth. The framework is not tied to KDE: any estimator providing $p_{0}\left(L,U\right)$ suffices; the kernel and bandwidth rule are specified in the case study.

##### FMEA-Guided Faulty Density

Faulty behavior is built from representative fault modes identified by failure mode and effects analysis (FMEA)—bias drift, gain variation, attenuation, stuck behavior, and similar deviations. With *M* such modes, the aggregated faulty density is modeled as a finite mixture [35]:

(27) $$f_{1}\left(s\right)=\sum_{\ell =1}^{M}\omega_{\ell }f_{1,\ell }\left(s\right),\;\sum_{\ell =1}^{M}\omega_{\ell }=1,\;\;\omega_{\ell }\geq 0,$$

where each $f_{1,\ell }\left(s\right)$ is obtained from fault-injection simulation, experiment, or engineering priors. The weights follow the FMEA failure rates: for the *r*-th single-fault case with rate $\lambda_{r}$,

(28) $$\omega_{r}=\frac{\lambda_{r}}{\sum_{q=1}^{R}\lambda_{q}},\;r=1,\ldots ,R,$$

such that $\omega_{r}$ is the conditional probability of that mode given one of the considered faults. Each $\lambda_{r}$ is a mode-level rate (component rate times mode frequency); identical components sharing a mode list are represented by a single entry whose rate reflects the instance count. The occurrence-weighted faulty KDE is

(29) $${\hat{f}}_{1}\left(s\right)=\sum_{r=1}^{R}\omega_{r}\frac{1}{N_{r}h_{r}}\sum_{m=1}^{N_{r}}K\;\left(\frac{s-s_{r,m}^{\left(1\right)}}{h_{r}}\right),$$

with bandwidth $h_{r}$ for case *r*; setting all $\omega_{r}=1/R$ recovers the equal-weight mixture. Per-mode results and grouped FMEA rates are reported in Appendix A and Appendix B, respectively.

In the present study, the FMEA-weighted faulty density is constructed for the operating condition used in the experiments. Within one confirmation horizon, the healthy and faulty violation probabilities are therefore treated as approximately constant. The following subsection selects the threshold band from the healthy operating envelope and then maps each mode-specific faulty density to its corresponding violation probability.

#### 3.2.2. Threshold Band and Single-Sample Violation Probabilities

The second workflow stage (Step 3 in Figure 1) maps the densities to the single-sample violation probabilities. The amplitude band contains the healthy operating range with a guard band rather than maximizing the healthy–faulty separation:

(30) $$L^{★}=s_{\min}^{\left(0\right)}-k_{g}{\hat{\sigma }}_{0},\;U^{★}=s_{\max}^{\left(0\right)}+k_{g}{\hat{\sigma }}_{0},$$

where $s_{\min}^{\left(0\right)}$ and $s_{\max}^{\left(0\right)}$ are the observed healthy extremes (the superscript (0) denotes the healthy condition $H_{0}$), ${\hat{\sigma }}_{0}$ the healthy standard deviation, and $k_{g}$ the guard factor. This keeps the empirical healthy violation rate near zero for the measured dataset; a larger $k_{g}$ suppresses false confirmations but lets more near-boundary faults stay inside the band. A statistically “optimal” rule—for example, a quantile threshold at a fixed nominal false-alarm level, or one minimizing the empirical false-alarm rate—would instead fix a non-zero per-sample violation probability $p_{0}$ under the healthy condition. For a latching, safety-critical confirmation monitor, a non-zero healthy violation probability is undesirable because the counter can accumulate such excursions. If the violations are temporally dependent, clustering could further increase the first-hitting probability, an effect not represented by the scalar Bernoulli model used here. The threshold would also become sensitive to the estimated density tail rather than being tied directly to the observed healthy envelope. Containing the measured healthy range with a guard band enforces $p_{0}\approx 0$ directly, is robust to density-estimation error in the tails, and shifts the entire false-confirmation burden onto the counter logic, where it is controlled analytically by the finite-horizon constraint. The separation-maximizing and explicit-$P_{FA}$ alternatives are nonetheless reported for comparison in Appendix B.2. For each candidate (L,U), $p_{0}\left(L,U\right)$ and $p_{1}\left(L,U\right)$ of Equations (9) and (10) are obtained by numerically integrating ${\hat{f}}_{0}$ and ${\hat{f}}_{1}$ outside the interval. The separation

(31) $$\Delta_{p}\left(L,U\right)=p_{1}\left(L,U\right)-p_{0}\left(L,U\right)$$

is reported only as a diagnostic, not the selection objective: maximizing $\Delta_{p}$ pushes the band into the healthy distribution, giving a large $p_{0}$ and severe false confirmations under the evaluated model. This mapping connects the distribution model to the counter reliability model: a too-narrow band raises $p_{0}$ and causes nuisance trips, whereas a too-wide band lowers $p_{1}$ and weakens fault sensitivity.

#### 3.2.3. Absorbing-DTMC Finite-Horizon Reliability Evaluation

The third workflow stage (Step 4 in Figure 1) evaluates the threshold-and-counter logic with the absorbing DTMC of Section 2.1. Given the parameter set Θ=(L,U,X,Y,H) (Equation (17)), the transition matrix $P_{i}\left(\Theta \right)$ is built from $p_{i}\left(L,U\right)$—$p_{0}$ for the healthy chain $P_{0}$ and $p_{1}$ for the faulty chain $P_{1}$—following Equation (6).

For an (H+1)-state counter, $P_{i}\left(\Theta \right)$ is (H+1)×(H+1); each non-absorbing row has at most two non-zero entries (the upward and recovery transitions) and the last row is the absorbing trip state:

(32) $$P_{i}\left(\Theta \right)=\left[\begin{array}{ccccc} {}* & * & 0 & \cdots & 0\\ {}* & 0 & * & \cdots & 0\\ \vdots & \vdots & \vdots & \ddots & \vdots \\ 0 & \cdots & * & 0 & *\\ 0 & \cdots & 0 & 0 & 1 \end{array}\right],$$

where * is a non-zero probability whose location depends on X,Y,H. The finite-horizon hitting probabilities are obtained by iterative state-probability propagation rather than dense matrix powers. From the initial distribution $\pi_{0}^{\left(i\right)}=e_{0}^{⊤}$,

(33) $$\pi_{k+1}^{\left(i\right)}=\pi_{k}^{\left(i\right)}\;P_{i}\left(\Theta \right),\;k=0,\ldots ,T_{h}-1,\;i\in \left\{0,1\right\}.$$

The finite-horizon false-confirmation and missed-detection probabilities (Equations (15) and (16)) are read from the absorbing-state mass of these propagated distributions: $e_{H}$ extracts the trip-state probability, which under $H_{0}$ is the false confirmation $P_{FA}\left(T_{h};\Theta \right)$ and under $H_{1}$ is the detection probability, whose complement is $P_{MD}\left(T_{h};\Theta \right)$. The average detection delay ADD(Θ) (Equation (14)) is obtained from the same propagation by tracking the first-hitting-time distribution of $\tau_{H}$ under $H_{1}$. Extremely small probabilities are reported on a logarithmic scale when needed. The DTMC propagation is verified against Monte Carlo simulation in Appendix C.2, while the computational benefit of reachable-state pruning is quantified in Appendix C.3.

#### 3.2.4. Counter Synthesis: Minimum-Delay Reliability-Constrained Objective

The fourth workflow stage (Step 5 in Figure 1) selects the counter parameters from the DTMC metrics. With the thresholds $\left(L^{★},U^{★}\right)$ fixed by Step 3, the counter parameters minimize the average detection delay subject to the finite-horizon reliability limits:

(34) $$\begin{array}{cc} \left(X^{★},Y^{★},H^{★}\right)=\underset{X,Y,H}{arg min}\; & \mathrm{ADD}(L^{★},U^{★},X,Y,H)\\ s.t.\;\; & P_{FA}\left(T_{h};L^{★},U^{★},X,Y,H\right)\leq \alpha ,\\ \; & P_{MD}^{\max,D}\left(T_{h};L^{★},U^{★},X,Y,H\right)\leq \beta ,\\ \; & X,Y,H\in N^{*},\;\;X\leq H,\;\;Y\leq H,\;\;H\leq H_{\max}, \end{array}$$

where α and β are the allowable false-confirmation and missed-detection probabilities, respectively. Here, $P_{MD}^{\max,D}$ denotes the maximum mode-wise missed-detection probability over the amplitude-detectable subset D. The $P_{FA}$ is evaluated at the worst tolerated noise level. In this work, $\alpha =10^{-9}$ is consistent with the catastrophic-level failure-probability objective in civil aircraft safety standards [36,37], here adopted as a conservative per-decision target rather than a per-flight-hour rate. The value $\beta =10^{-6}$ is adopted as a stringent mode-conditional target for the amplitude-detectable subset D. For the multi-mode fault population, the final configuration is verified against the worst-case per-mode probability $P_{MD}^{\max,D}\leq \beta$. Fault modes whose signatures remain within the selected amplitude band are not included in the β guarantee. The method applies to any user-specified (α,β). This minimum-delay constrained form mirrors the classical sequential-detection criterion of minimizing detection delay under a false-confirmation constraint. When explicit limits α,β are unavailable, the same metrics can instead be combined into a risk-weighted objective

(35) $$J\left(\Theta \right)=w_{FA}\;P_{FA}\left(T_{h};\Theta \right)+w_{MD}\;P_{MD}\left(T_{h};\Theta \right),\;w_{FA},w_{MD}\geq 0,\;w_{FA}+w_{MD}>0,$$

which is retained as the general formulation. The resulting optimized parameter set is $\Theta^{★}=\left(L^{★},U^{★},X^{★},Y^{★},H^{★}\right)$, as shown at the bottom of Figure 1.

Although Equation (34) admits a joint search over thresholds and counter parameters, the synthesis is implemented in two stages for interpretability and consistency with engineering threshold selection. In the first stage, $\left(L^{★},U^{★}\right)$ are fixed by the containment rule of Equation (30). In the second stage, the integer-valued (X,Y,H) are enumerated by grid search over $\Omega_{\mathrm{cum}}$ (Equation (18)); for each candidate, $P_{0}$ and $P_{1}$ are constructed from Equation (6), the finite-horizon metrics are evaluated by the state propagation of Equation (33), and the configuration minimizing ADD subject to the constraints of Equation (34) is selected. The KDE settings, threshold grids, and search ranges for X,Y,H are given in the case study. Because density estimation, probability mapping, DTMC evaluation, and the parameter search are all offline, the online monitor remains lightweight.

### 3.3. Online Deployment and Summary

All computationally intensive operations—density construction, probability mapping, DTMC evaluation, and parameter search—are performed offline, so the online monitor preserves the original threshold-and-counter structure without runtime distribution estimation or retraining. Once $\Theta^{★}$ is obtained, each sampling instant requires only: (i) evaluating whether $s_{k}\notin \left[L^{★},U^{★}\right]$; (ii) a saturated integer counter update; and (iii) tripping and latching if $C_{k}=H^{★}$. The online complexity is therefore O(1) per sample with constant memory, as only the current counter value is stored.

In summary, the healthy and faulty signal distributions are estimated from measured, simulated, and fault-injection data and mapped to $p_{0},p_{1}$; the amplitude band contains the healthy envelope with a guard band, keeping the measured healthy violation rate near zero; and the counter parameters are then optimized by minimizing conditional ADD subject to finite-horizon false-confirmation and missed-detection constraints. All synthesis is offline, leaving a lightweight constant-time online monitor.

## 4. Experiments

This section validates the proposed method on an LVDT sum-voltage monitor, using real aircraft healthy measurements and Simulink-based fault injection. The paired healthy measured and simulated data are used to identify an affine calibration mapping. Representative faults are first injected into the Simulink model, and the mapping is subsequently applied to the resulting faulty outputs. From these data, we build the healthy and occurrence-weighted faulty signal distributions, select the amplitude band and synthesize the up/down-counter parameters, and evaluate the resulting monitor against representative baselines and across a range of noise levels.

The remainder of this section covers the experimental setup (Section 4.1), data processing and signal-distribution construction (Section 4.2), performance evaluation (Section 4.3), and robustness evaluation (Section 4.4); per-mode fault coverage is reported in Appendix A, data and threshold validation in Appendix B, and numerical verification and search-efficiency results in Appendix C.

**Figure 2:**
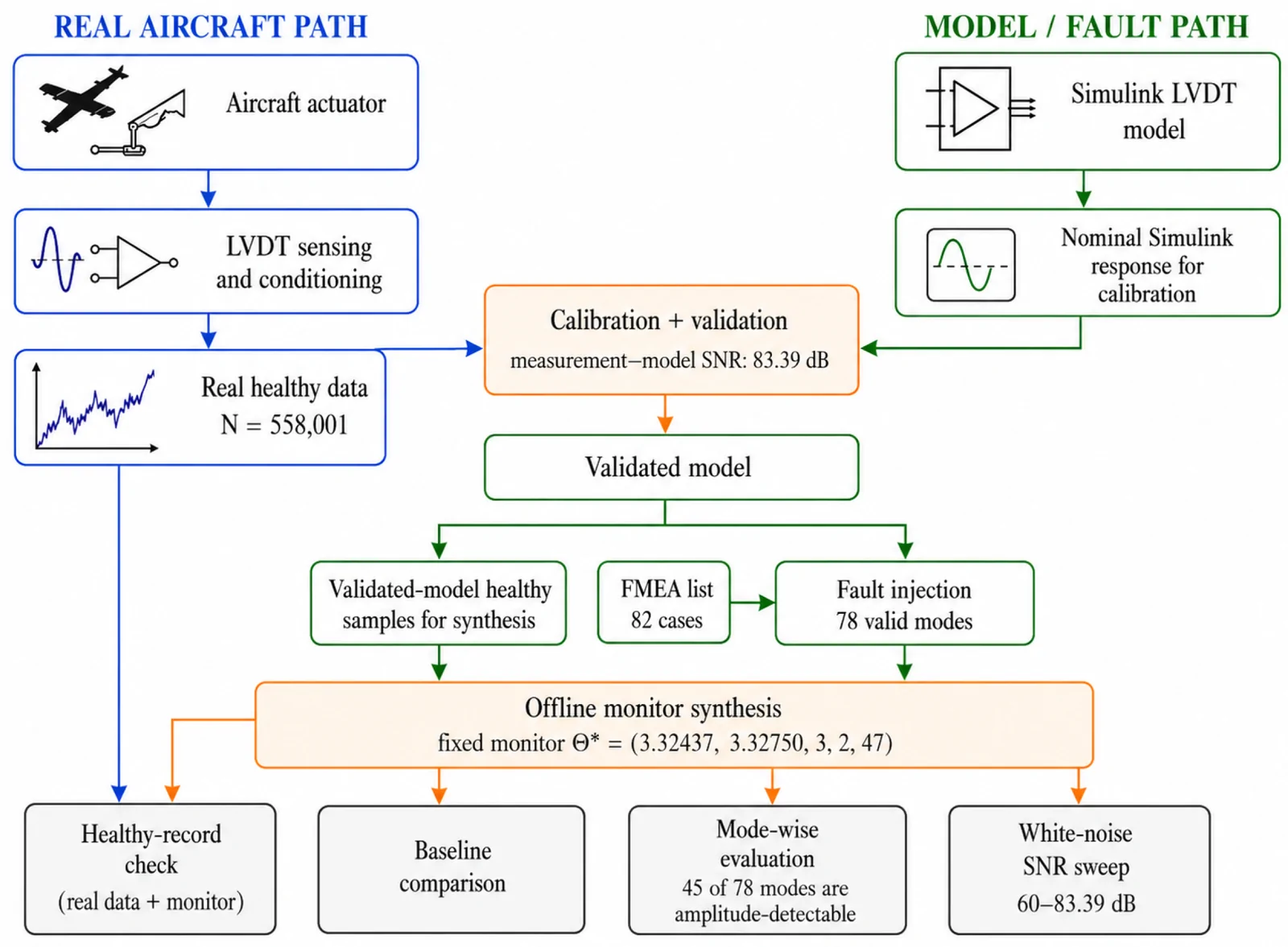
Experimental context and data flow.

Figure 2 summarizes the experimental context and the data flow from real aircraft measurements and the validated Simulink model to offline monitor synthesis and evaluation.

### 4.1. Experimental Setup

The monitored signal is the demodulated sum voltage of a Linear Variable Differential Transformer (LVDT), a position-feedback sensor widely used in aerospace actuation and flight-control systems. In this electro-mechanical actuation channel, the LVDT senses the mechanical displacement of the control surface and feeds it back to the flight-control loop, so confirming faults in its sensing circuit is essential to the reliable mechanical attitude control of the aircraft. Its key operating parameters and monitoring logic are:

- Excitation: the LVDT is driven by a 7.00±5%Vrms, 1953±5%Hz excitation.
- Sampling: the sum voltage is sampled at 24kHz (about 12 samples per excitation period); its definition follows Equation (1).
- Monitoring logic: the sum voltage is compared against prescribed lower and upper amplitude thresholds; rather than confirming a fault on a single violation, the comparison result feeds an up/down counter, and a fault is declared only when the accumulated evidence reaches the trip limit.

The experiments draw on three data sources:

- Simulink healthy data: clean nominal sum-voltage responses generated by the LVDT model under healthy operation.
- Real healthy data: demodulated sum-voltage measurements collected from the aircraft, used to validate that the Simulink model reproduces the nominal behavior and to characterize the aggregate measurement–model discrepancy level.
- Faulty data: representative faulty samples generated by fault injection in the validated model, since controlled physical fault experiments on the LVDT are impractical; the injected modes are aggregated into a single faulty distribution weighted by their FMEA occurrence probabilities.

Once the model is validated against the real measurements, the calibrated Simulink model supplies both the healthy and faulty samples used for threshold selection and counter-parameter optimization.

### 4.2. Data Processing

#### 4.2.1. Data Processing and Signal Distribution Construction

##### Healthy Data

For the healthy condition, two complementary data sources are used. First, real healthy data were collected from the actual LVDT on the aircraft. Second, a Simulink model of the same LVDT circuit was built and used to generate healthy responses. The measured data ground the analysis in real operating behavior, while the model provides clean, abundant samples; aligning the two also lets us check that the model reproduces the real signal.

These two sources are processed in three steps:

- Alignment and calibration: because the measured and simulated signals may use different sampling grids, they are aligned over their common time interval, the Simulink signal is interpolated onto the measurement grid, and an affine gain-bias calibration removes systematic mismatch (Section Data Preparation and Controlled Noise Modeling).
- Validation: comparing the calibrated simulation output with the measured signal gives an aggregate measurement–model discrepancy that includes measurement noise, residual model mismatch, and interpolation and alignment errors. The resulting calibration SDR is 85.48dB (Equation (22)). Detailed results on the calibration accuracy and model-order comparison are reported in Appendix C.1.
- Sampling for KDE: the validated, gain-calibrated model is used as the source of the healthy samples $D_{0}={\left\{s_{k}^{\left(0\right)}\right\}}_{k=1}^{N_{0}}$ characterizing $H_{0}$; to reduce cost and temporal redundancy, the Simulink record is uniformly subsampled to 50,000 points, with the selected band insensitive to this choice (Appendix B.3). For controlled sensitivity studies, additional noisy data are generated by Equations (24) and (25).

##### Faulty Data

Representative FMEA-guided faults are first injected into the Simulink model, and the resulting faulty outputs are then calibrated in the same manner as the healthy Simulink output. The campaign is built up as follows:

- Components: 30+ components, each with two or three representative fault modes;
- Cases: 80+ candidates, of which 78 produce valid time series;
- Coverage: faults span the sensing element, signal-conditioning, excitation/demodulation, and wiring/connector paths.

Rather than treating these 78 modes equally, each single-fault case is weighted by its FMEA failure rate, normalized by Equation (28). The aggregated faulty density ${\hat{f}}_{1}\left(s\right)$ is therefore the distribution conditioned on one of the considered single faults occurring, with rare modes contributing less and common modes more. Per-mode results and grouped FMEA rates are reported in Appendix A and Appendix B, respectively.

We estimate the probability densities of both the healthy and faulty signals by KDE (Gaussian kernel, Scott’s rule; Section 3.2.1). The sample counts and signal ranges of the two healthy sources and the 78-mode faulty set are listed in Table 1. We report the signal range rather than a single mean and standard deviation because the faulty condition aggregates 78 fault modes into a multi-modal distribution, for which a mean and standard deviation would be misleading.

#### 4.2.2. Experimental Estimation of Single-Sample Violation Probabilities

The KDE densities give the single-sample violation probabilities $p_{0}\left(L,U\right)$ and $p_{1}\left(L,U\right)$ (Equations (9) and (10)). To check the healthy estimate, the threshold selection is repeated independently on the two healthy sources, summarized in Table 2. The selected thresholds are highly consistent across sources ($|\Delta L^{★}|=3.8\;\times \;10^{-4}$, $|\Delta U^{★}|=2.4\;\times \;10^{-4}$), and the residual difference in $p_{1}$ reflects the sensitivity of the faulty violation probability to small threshold shifts.

**Figure 3:**
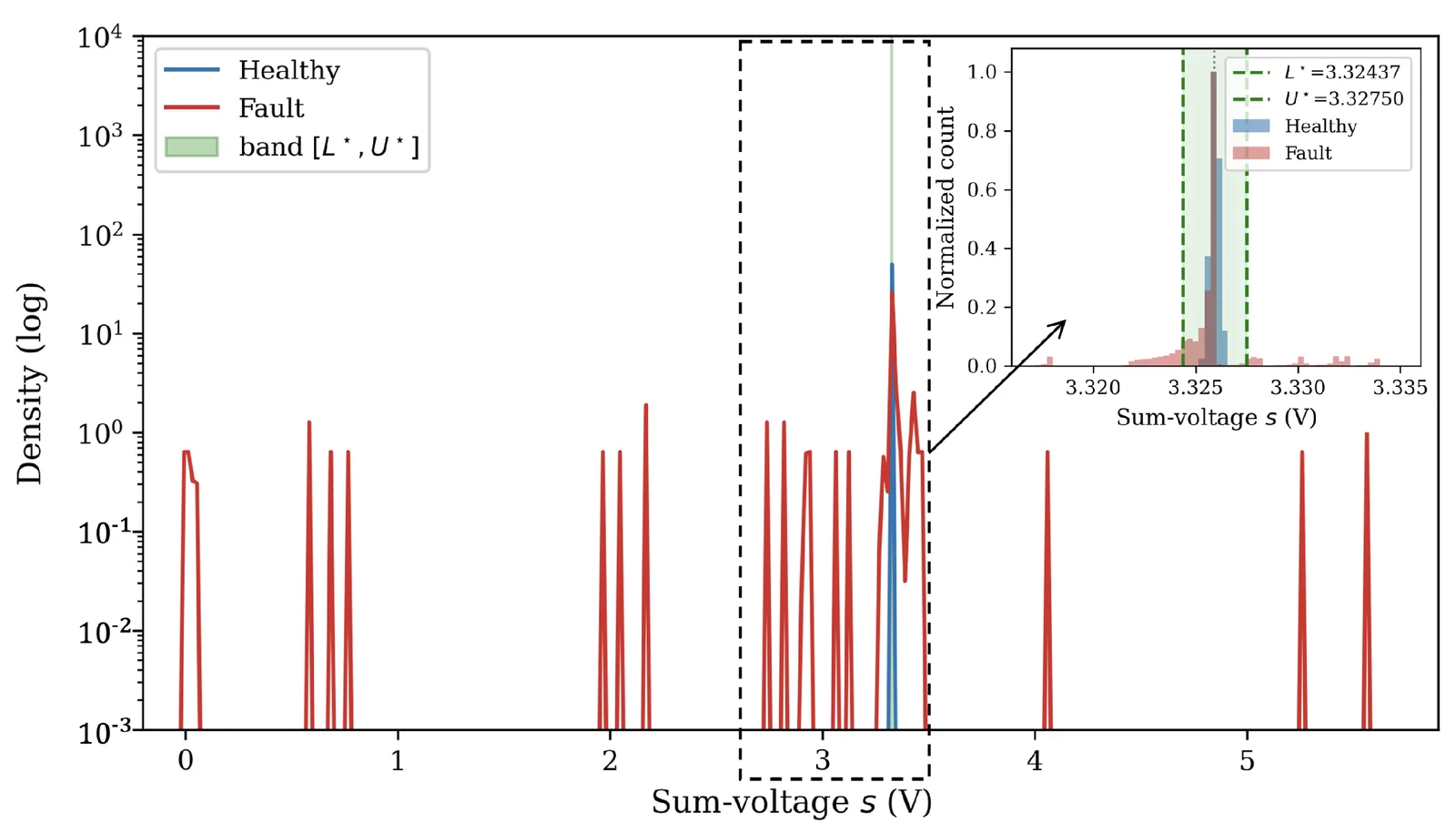
Measured healthy and Simulink fault-injection sum-voltage distributions with the $3{\hat{\sigma }}_{0}$ containment band. The main panel shows the full signal range, including the separated open- and short-circuit modes, whereas the inset enlarges the near-nominal region and shows the healthy distribution, containment band, and overlapping fault modes.

Figure 3 shows the KDE-estimated healthy and occurrence-weighted faulty densities. The overlap between ${\hat{f}}_{0}\left(s\right)$ and ${\hat{f}}_{1}\left(s\right)$ explains why a single amplitude band cannot detect every simulated fault mode without raising the false-confirmation risk; the band therefore prioritizes healthy-data containment, with the remaining fault coverage quantified in Appendix A. The overlap between the healthy and faulty distributions directly affects detection performance. Fault probability mass that remains inside the containment band $\left[L^{★},U^{★}\right]$ produces no threshold violation and is therefore indistinguishable from healthy operation, increasing $P_{MD}$. In contrast, probability mass outside the band increases the single-sample violation probability $p_{1}$ and reduces $P_{MD}$. Thus, widening the band suppresses false confirmations but may reduce sensitivity to near-boundary faults.

### 4.3. Performance Evaluation

The offline synthesis proceeds in two stages: the first fixes the amplitude thresholds $L^{★},U^{★}$ from the predefined engineering ranges, and the second optimizes the counter parameters X,Y,H using the finite-horizon reliability metrics. The complete parameter set Θ is defined in Equation (17), and the monitoring horizon is $T_{h}$. The single violation probabilities $p_{0}\left(L,U\right)$ and $p_{1}\left(L,U\right)$ are obtained from the KDE estimates of Section 4.2.1. For each candidate counter, we use the absorbing DTMC (Equation (33)) to evaluate its reliability metrics. For the up/down counters, the ADD is computed separately: detected faults contribute their average detection delay, while faults not detected within $T_{h}$ appear instead in the missed-detection probability $P_{MD}\left(T_{h}\right)$. The search space and numerical settings are summarized in Table 3.

In the first stage, the amplitude thresholds are fixed by the healthy-envelope containment rule (Equation (30), guard factor $k_{g}=3$), giving $L^{★}=3.32437$ and $U^{★}=3.32750$. These thresholds keep healthy samples inside the admissible band (empirically $p_{0}\approx 0$; see Appendix B.4) and are shared by all monitors below. We adopt this containment rule for robustness rather than to maximize separation because the guard margin reduces sensitivity to healthy excursions and to errors in the estimated distribution tails. Alternative threshold policies—maximizing $p_{1}-p_{0}$ or imposing an explicit $P_{FA}$ constraint—are compared in Appendix B.2.

With $L^{★},U^{★}$ fixed, the confirmation logics differ only in how many counter parameters they must search:

- Threshold-only monitor: no search; it trips immediately once $s_{k}\notin \left[L^{★},U^{★}\right]$.
- Delay-timer baseline: searches only the consecutive-violation count *m*, giving m=14.
- Unit-step up/down counter: fixes X=1,Y=1 and searches only the trip limit *H*, giving H=15.
- Proposed method: searches (X,Y,H) jointly under Equation (34), giving $\left(X^{★},Y^{★},H^{★}\right)=\left(3,2,47\right)$.

**Figure 4:**
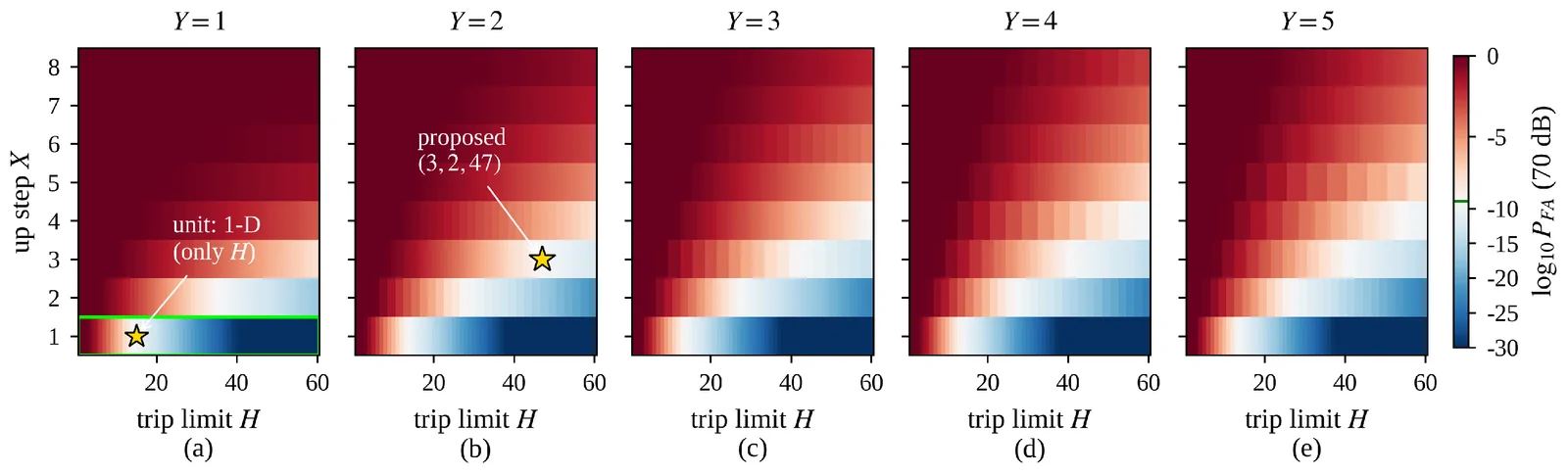
False-confirmation probability ($P_{\mathrm{FA}}$) over the (X,H) plane under 70dB noise: (**a**) (Y=1); (**b**) (Y=2); (**c**) (Y=3); (**d**) (Y=4); and (**e**) (Y=5). Blue regions satisfy $P_{\mathrm{FA}}\leq \alpha$, whereas red regions violate this constraint. The stars in panels (**a**,**b**) mark the unit-step counter (1, 1, 15) and the proposed configuration (3, 2, 47), respectively.

Figure 4 shows the feasibility of the joint (X,Y,H) search at 70dB: each panel presents the false-confirmation probability over the (X,H) plane at a fixed *Y*, with blue regions satisfying $P_{FA}\leq \alpha$ and red regions violating this constraint. The marked unit-step counter (1, 1, 15) and the proposed configuration (3, 2, 47) both satisfy the false-confirmation constraint. Among all feasible candidates, the proposed configuration is selected because it attains the smallest detection delay.

**Figure 5:**
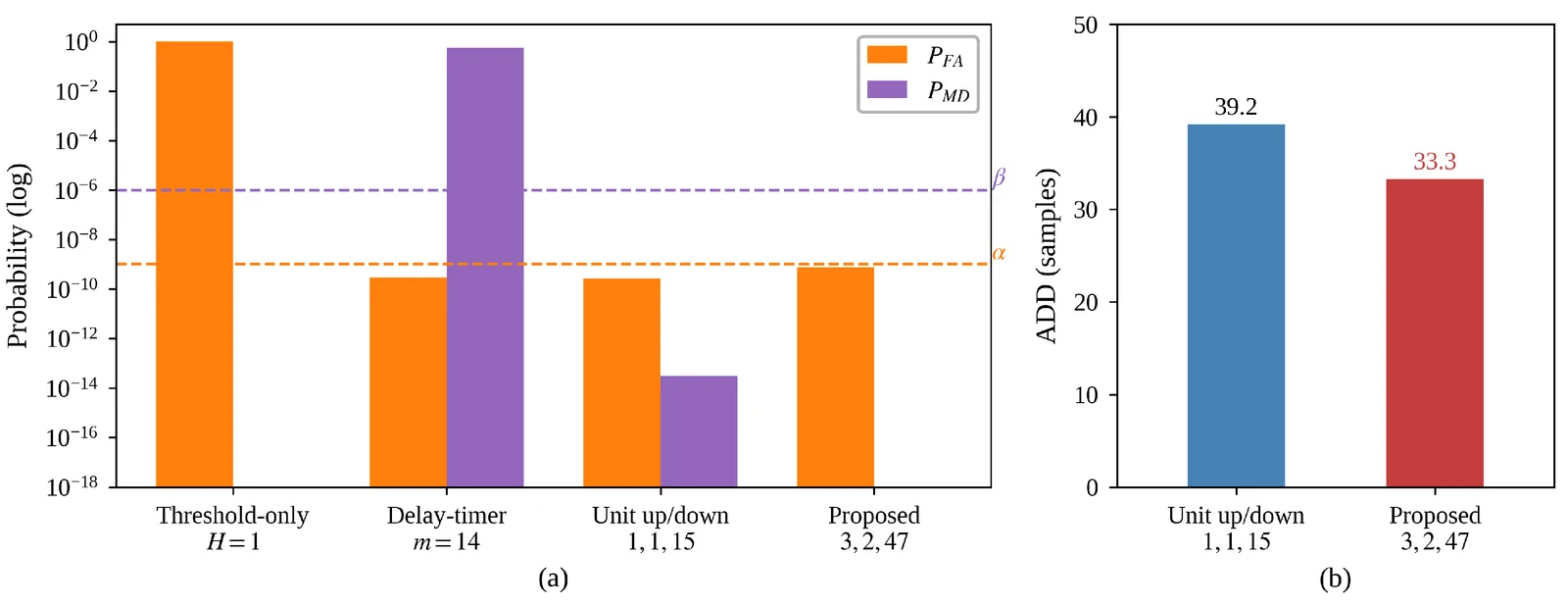
Monitor comparison on an incipient near-boundary fault under 70dB noise. (**a**) False-confirmation and missed-detection probabilities relative to α and β. (**b**) Detection delay of the two feasible up/down counters.

All four monitors are evaluated on the same incipient near-boundary open-circuit mode under identical conditions—the shared threshold band $\left[L^{★},U^{★}\right]$, the design noise level ${\mathrm{SNR}}_{\mathrm{design}}=70\;\mathrm{dB}$ (giving a healthy violation rate $p_{0}=0.14$ and a representative per-sample violation rate $p_{1}=0.68$ at this near-boundary operating point), and the monitoring horizon $T_{h}=500$—against the false-confirmation and missed-detection limits $\alpha =10^{-9}$ and $\beta =10^{-6}$ (the maximum tolerated $P_{FA}$ and $P_{MD}$). We deliberately choose this near-boundary fault: its output fluctuates around the healthy region, so the violations are intermittent and far more challenging for the counters than gross open/short faults, which every monitor confirms easily. The resulting metrics are summarized in Table 4 and visualized in Figure 5.

As Table 4 shows, the threshold-only monitor violates the false-confirmation constraint ($P_{FA}=1.00$): with no confirmation logic, every noise-induced excursion trips it immediately. The delay timer satisfies α but misses many flickering faults ($P_{MD}=0.486$), because a single in-band sample resets its consecutive count and the intermittent violations rarely accumulate to m=14. Only the two up/down counters satisfy both the false-confirmation and missed-detection constraints; among them, the proposed asymmetric configuration (3, 2, 47) attains the smaller detection delay (ADD=33.3 vs. 39.2 samples). The asymmetry is what drives this: the upward step $X^{★}>Y^{★}$ accumulates quickly on persistent violations, while the recovery step $Y^{★}$ drains isolated noise-induced violations.

Figure 5 provides a visual summary of the feasibility margins and detection-delay comparison reported numerically in Table 4. Because Table 4 and Figure 5 use one representative near-boundary fault, the missed-detection guarantee was additionally verified mode by mode over the 78 simulated fault modes. Among them, 45 modes are amplitude-detectable, and all satisfy $P_{MD,\ell }<10^{-15}$. The worst detectable mode is D08, with $p_{1,\ell }=0.6403$ and $P_{MD,\ell }=7.8\times 10^{-16}$, still well below $\beta =10^{-6}$. The remaining 33 modes stay within the selected amplitude band and therefore fall outside the β-guarantee scope of the amplitude-based monitor. Detailed per-mode results are reported in Appendix A.

### 4.4. Robustness Evaluation

**Figure 6:**
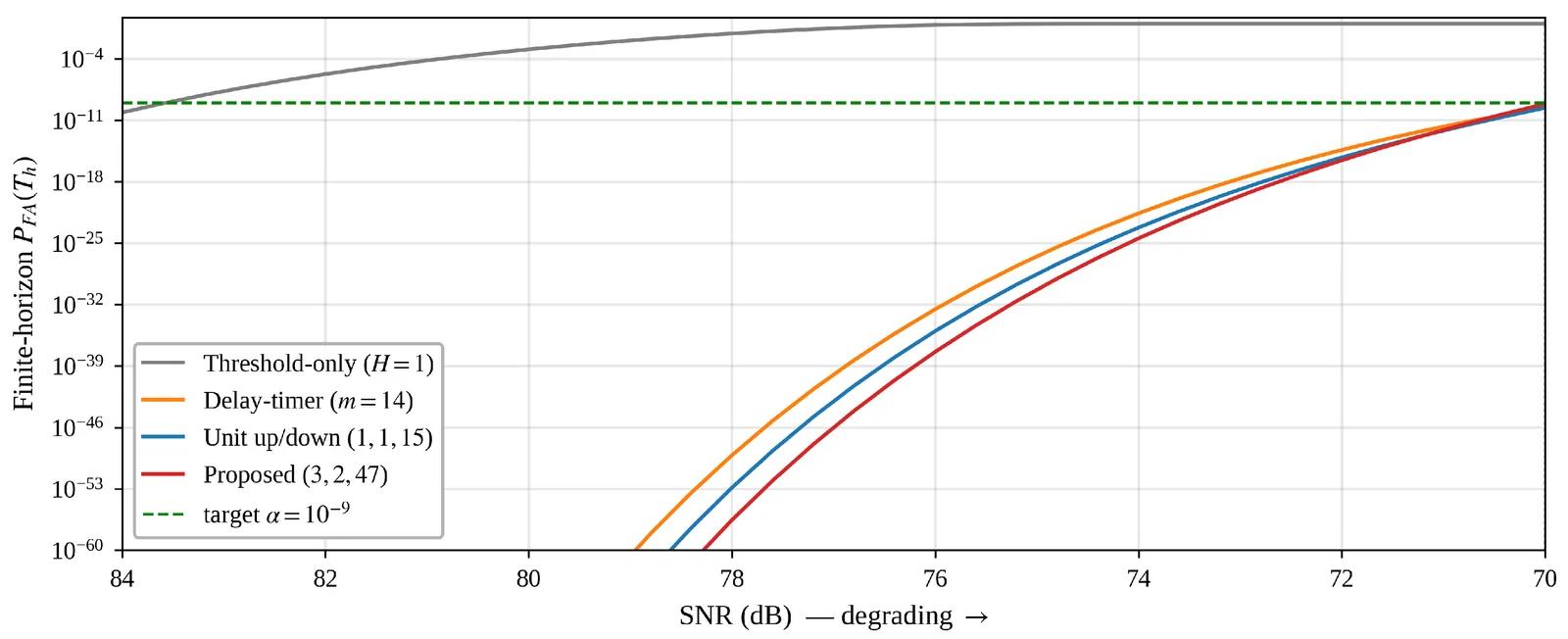
Finite-horizon false-confirmation probability versus SNR on a logarithmic scale. The threshold-only monitor exceeds the target α even near the nominal SNR, whereas both up/down counters remain below α down to 70dB.

Robustness is evaluated by applying the fixed optimized monitor $\Theta^{★}$ under additional white Gaussian noise, without re-optimizing the parameters at each SNR. The measured signal quality is 83.39dB, and the design SNR is set to 70dB, about 13dB below the measured level. Lower-SNR tests at 65dB and 60dB are included to check whether the fixed configuration remains feasible below the design point. Figure 6 compares the false-confirmation robustness of all four monitors across the tested SNR values, whereas Table 5 reports the corresponding numerical metrics for the fixed optimized monitor.

The fixed monitor remains within the false-confirmation target from the nominal 83.39dB case down to 70dB. At 65dB and 60dB, the constraint is violated by a large margin. Thus, 70dB is the lowest tested SNR at which the selected monitor (3, 2, 47) satisfies the target under the white-noise model.

The reported SNR results are conditional on the independent Bernoulli abstraction used by the DTMC and on the controlled white-Gaussian-noise stress model. They therefore characterize robustness under the specified independent-noise assumption. Temporal dependence in real measurement sequences is outside the scope of the present experiment and is left for future work.

## 5. Discussion

### 5.1. Application Methodology and Implementation Path

The proposed framework is intended as a deployable design procedure rather than a one-off analysis. In practice, it is applied in four offline steps that a monitor designer can follow directly:

(i) Collect paired healthy measured and simulated data, identify the affine calibration mapping, and estimate the healthy density ${\hat{f}}_{0}$ from the calibrated healthy Simulink output (Section Healthy Density Estimation).
(ii) Inject the FMEA-guided fault cases into the Simulink model, calibrate each resulting faulty output in the same manner as the healthy Simulink output, and estimate the mode-specific faulty densities ${\hat{f}}_{1,\ell }$. The FMEA-weighted density ${\hat{f}}_{1}$ is constructed only for population-level distribution and occurrence-weighted coverage analysis (Section FMEA-Guided Faulty Density).
(iii) Fix the amplitude band $\left(L^{★},U^{★}\right)$ using the healthy-envelope containment rule.
(iv) Calculate $p_{0}$ and the mode-specific violation probabilities $p_{1,\ell }$, and synthesize the counter parameters $\left(X^{★},Y^{★},H^{★}\right)$ through the minimum-delay constrained search, with the missed-detection constraint imposed on $P_{MD}^{\max,D}$ in Equation (34).

The designer specifies the reliability targets (α,β), the horizon $T_{h}$, the guard factor $k_{g}$, the design SNR, and the counter search ranges. Because all computationally intensive operations are performed offline, the deployed monitor requires only a threshold comparison and an integer counter update per sample, with O(1) online time and memory. The procedure can therefore be retrofitted to existing threshold-and-counter monitors without changing their online execution structure.

### 5.2. Performance Trade-Offs and Mechanism Interpretation

The comparison results clarify why the asymmetric up/down counter is more effective for the considered near-boundary fault. The threshold-only monitor reacts immediately to isolated excursions and therefore violates the false-confirmation requirement, whereas the delay timer discards all accumulated evidence whenever one in-band sample occurs, resulting in a high missed-detection probability for intermittent violations. In contrast, the proposed configuration $X^{★}>Y^{★}$ accumulates persistent out-of-band evidence faster than it removes it, while still allowing isolated excursions to decay. This mechanism explains why the proposed counter satisfies both reliability constraints and reduces ADD from 39.2 to 33.3 samples relative to the unit-step counter.

The results also reveal an inherent trade-off between false-confirmation robustness and amplitude-based fault coverage. The healthy-envelope containment rule suppresses healthy violations, but fault modes whose signatures remain within the same band cannot be separated by amplitude alone. Consequently, 45 of the 78 simulated modes are amplitude-detectable, corresponding to a per-case coverage of 57.7% and an occurrence-weighted coverage of 34.0%. These coverage values characterize a limitation of the selected observable rather than a failure of the confirmation counter. The white-noise sweep further identifies 70dB as the lowest tested SNR at which the fixed configuration satisfies the false-confirmation constraint under the assumed independent-noise model. The violation of this constraint at 65dB and 60dB defines the tested operating boundary of the design.

### 5.3. Sensor Errors, Scope and Limitations

Sensor errors are not modeled as a separate probability distribution in the present study. Representative faults are first injected into the Simulink model, and the resulting faulty outputs are then calibrated in the same manner as the healthy Simulink output. The calibrated healthy and faulty outputs are used for distribution construction and monitor parameter evaluation.

The results assume that the calibration mapping identified under healthy operation remains applicable to the simulated faulty outputs. Random sensor errors, unit-to-unit variation, temperature drift, ADC uncertainty, and changes in the calibration relationship are outside the present scope.

The amplitude-and-counter monitor has an amplitude-based detection scope. Fault modes whose steady signatures remain within the selected healthy band cannot be distinguished by this monitor and require complementary observables, such as excitation, current, or built-in-test signals. The present study also assumes local stationarity and independent threshold violations during one confirmation horizon. Condition-dependent distributions, temporally correlated violations, and detailed fault-evolution models remain subjects for future work.

## 6. Conclusions

This paper presented a reliability-oriented parameter synthesis workflow for threshold-and-counter fault-confirmation monitoring, validated on an LVDT sum-voltage monitor. It replaces conventional heuristic tuning with a quantitative procedure that synthesizes the thresholds and counter parameters directly from finite-horizon reliability metrics, while leaving the deployed online monitor lightweight. The main contributions are:

1. End-to-end reliability-oriented workflow: FMEA-guided distribution modeling, finite-horizon reliability evaluation, and offline synthesis are combined into one workflow, replacing heuristic counter tuning with a quantitative configuration.
2. Finite-horizon DTMC reliability evaluation: the threshold-and-counter logic is modeled as an absorbing DTMC, giving the exact finite-horizon $P_{FA}$, $P_{MD}$, and ADD at $O(T_{h}\left(H+1\right))$ cost, instead of $2^{T_{h}}$ enumeration or long-run rates.
3. Two-stage offline synthesis with lightweight deployment: thresholds and counter parameters are searched offline, leaving an online monitor that runs in O(1) per sample, independent of the design search.

In the LVDT case study, the synthesized configuration (3, 2, 47) raised no false confirmation over 558,001 measured healthy samples and held $P_{FA}\leq 10^{-9}$ even when added white noise lowered the SNR to 70dB—about 13dB below the measured 83.39dB. Among the compared confirmation logics, it was the only one to meet both the false-confirmation and missed-detection targets while attaining the smallest detection delay. Future work will study additional operating conditions and correlated-input reliability models where the healthy violation rate is non-negligible.   

## Figures and Tables

**Table 1:** Summary of healthy and occurrence-weighted faulty signal samples.

Condition	Number of Samples/Modes	Signal Range
Healthy $H_{0}$ (real)	$N_{\mathrm{real}}=558,001$	[3.3250,3.3268]
Healthy $H_{0}$ (sim)	$N_{\mathrm{sim}}=8,061,518$	[3.3247,3.3271]
Faulty $H_{1}$	78 modes (of 82)	$\left[7.36\times 10^{-9},\;5.5728\right]$

**Table 2:** Cross-source consistency of the healthy KDE estimates and synthesized thresholds. The superscript ★ denotes a quantity selected by the offline synthesis procedure.

Quantity	Value
Bandwidth $h_{0}$ (real)	$1.60\times 10^{-5}$
Bandwidth $h_{0}$ (Simulink)	$2.64\times 10^{-5}$
$|\Delta L^{★}|$	$3.8\times 10^{-4}$
$|\Delta U^{★}|$	$2.4\times 10^{-4}$
$|\Delta p_{0}|$	≈0
$|\Delta p_{1}|$	$1.2\times 10^{-3}$

**Table 3:** Search space and numerical settings for offline parameter synthesis.

Parameter	Candidate Range/Setting
*X*	X={1,2,…,50}
*Y*	Y={1,2,…,5}
*H*	H={1,2,…,100}
$T_{h}$	500 samples
${\mathrm{SNR}}_{\mathrm{design}}$	70dB
α,β	$\leq 10^{-9},\;\leq 10^{-6}$

**Table 4:** Monitor comparison on an incipient near-boundary fault.

Method	Searched Params	$P_{\mathrm{FA}}\left(T_{h}\right)$	$P_{\mathrm{MD}}\left(T_{h}\right)$	ADD
Threshold-only monitor	none	1.00	≈0	–
Delay-timer baseline	m=14	$3.66\times 10^{-10}$	0.486	–
Unit-step up/down counter	H=15	$3.24\times 10^{-10}$	$5.6\times 10^{-16}$	39.2
Proposed method	X,Y,H=3,2,47	$9.37\times 10^{-10}$	$6.7\times 10^{-16}$	33.3

**Table 5:** Robustness of the fixed optimized monitor $\Theta^{★}$ across SNR ($T_{h}=500$). $P_{FA}$ and $p_{0}$ are evaluated under the healthy condition; ADD depends only on the faulty-side rate $p_{1}=0.68$ and is therefore constant across SNR. Rows with $P_{FA}\gg \alpha$ are infeasible operating points.

SNR (dB)	$p_{0}$ (Noise)	$P_{\mathrm{FA}}\left(T_{h}\right)$	$P_{\mathrm{MD}}\left(T_{h}\right)$	ADD
83.4 (nominal)	≈0	$7.5\times 10^{-177}$	≤β	33
80	≈0	$2.1\times 10^{-86}$	≤β	33
75	$8.4\times 10^{-3}$	$6.3\times 10^{-31}$	≤β	33
70	$1.4\times 10^{-1}$	$9.4\times 10^{-10}$	≤β	33
65	$4.0\times 10^{-1}$	$7.7\times 10^{-1}\;\left(\gg \;\alpha \right)$	≤β	33 (infeasible)
60	$6.4\times 10^{-1}$	≈1.0(≫α)	≤β	33 (infeasible)

## Data Availability

The data presented in this study are available from the corresponding author upon reasonable request. The data are not publicly available because they contain proprietary aircraft test information.

## Abbreviations

ADC	Analog-to-Digital Converter
ADD	Average Detection Delay
DTMC	Discrete-Time Markov Chain
FAR	False-Alarm Rate
FMEA	Failure Mode and Effects Analysis
i.i.d.	Independent and Identically Distributed
KDE	Kernel Density Estimation
LVDT	Linear Variable Differential Transformer
MAR	Missed-Alarm Rate
$P_{FA}$	False-Confirmation Probability
$P_{MD}$	Missed-Detection Probability
SDR	Signal-to-Discrepancy Ratio
SNR	Signal-to-Noise Ratio

## Appendix A. Fault Detection Coverage and Per-Mode Detectability

The containment band balances fault coverage against false-confirmation robustness. A fault is classified as amplitude-detectable only when the steady-state median of its sum voltage lies outside $\left[L^{★},U^{★}\right]=\left[3.32437,3.32750\right]$, as defined in Section 4.3. The evaluation includes the same 78 fault modes used to construct ${\hat{f}}_{1}\left(s\right)$, as described in Section 4.2.1. Fault categories are derived from the component-level fault list. Table A1 summarizes the detection coverage, and Table A2 provides the mode-specific results. The occurrence weight $\omega_{r}$ for each mode is calculated using Equation (28).

Per case, 57.7% (45/78) of the modes are detectable (Table A1): nearly all short-circuit and most open-circuit modes leave the band, whereas most parameter-drift modes stay near nominal and are amplitude-indistinguishable from healthy operation. The FMEA-weighted coverage drops to 34.0%, driven by the two power transistors (56.3% combined occurrence) whose modes are not amplitude-separable. The FMEA nominal allocation of 97.8% is a channel assignment, not an empirical detectability figure; the 34.0% is the subset separable under the false-confirmation requirement. Near-nominal modes require complementary monitors (excitation, current, BIT). A few modes lie within $10^{-4}$ of a band edge, so their detectability is sensitive at that level—the same near-boundary regime that produces the flickering incipient faults central to the counter’s advantage.

**Table A1:** Fault detection coverage under the selected threshold band, over the 78 valid simulated modes. Detectability is judged on each mode’s steady median against $\left[L^{★},U^{★}\right]$; every mode is itemized in Table A2.

Fault Type	Detectable/Total	Note
Short circuit	18/18	all leave the admissible band
Open circuit	16/26	most drive the sum voltage far off; a few stay near nominal
Parameter drift	11/34	most settle at ≈ nominal
Per-case total	45/78(57.7%)	each valid mode counted once
Occurrence-weighted coverage	34.0%	same FMEA weights as the faulty-distribution construction
FMEA nominal allocation	97.8%	FMEA assignment to sum-voltage monitoring; not estimated from the 78 modes

### Per-Mode Missed-Detection

The missed-detection guarantee $P_{MD}\leq \beta$ is stated over the amplitude-detectable subset D (|D|=45); the 33 modes whose steady median lies inside $\left[L^{★},U^{★}\right]$ are, by construction, not separable by any amplitude threshold and lie outside the detection scope of an amplitude-based monitor rather than being a failure of the counter. To confirm that the requirement holds for every detectable mode—not only the representative near-boundary case of Table 4—the per-mode missed-detection probability was evaluated at the deployed $\Theta^{★}=\left(3,\;2,\;47\right)$ using each mode’s own $p_{1,\ell }$ (from its steady samples against the band). All 45 detectable modes satisfy $P_{MD,\ell }<10^{-15}$, i.e. more than nine orders of magnitude below $\beta =10^{-6}$, and the worst single mode is the near-boundary drift mode D08 ($p_{1}=0.640$, the smallest per-sample violation probability among the detectable modes), still with $P_{MD}<10^{-15}$. Evaluated at this worst mode, the proposed counter attains ADD=38.5 samples, against 49.0 for the unit up/down counter, so it remains the fastest feasible confirmation logic under the most adversarial detectable mode as well as under the representative fault of Table 4.

**Table A2:** The 78 valid fault-injection modes: recovered type, steady median (real scale), normalized FMEA weight $\omega_{r}$ (%), and amplitude detectability (Y = outside the band). Displayed weights are rounded to two decimals; exact weights sum to 100% and detectable weights to 34.0%. Mode labels use (D), (O), and (S) prefixes for the drift, open-circuit, and short-circuit categories, respectively.

Mode	Type	Median	$\omega_{r}$ (%)	Det.	Mode	Type	Median	$\omega_{r}$ (%)	Det.
D01	Drift	4.06105	0.19	Y	O06	Open	3.36686	0.28	Y
D02	Drift	3.27892	0.19	Y	O07	Open	3.34803	0.28	Y
D03	Drift	3.12223	0.09	Y	O08	Open	3.34863	0.28	Y
D04	Drift	2.94912	0.19	Y	O09	Open	3.31777	0.28	Y
D05	Drift	3.32429	0.19	Y	O10	Open	3.32580	0.28	–
D06	Drift	3.32580	0.19	–	O11	Open	3.32580	0.28	–
D07	Drift	3.32560	0.19	–	O12	Open	3.32581	0.28	–
D08	Drift	3.32763	0.19	Y	O13	Open	3.32580	0.28	–
D09	Drift	3.33234	0.19	Y	O14	Open	3.32582	0.28	–
D10	Drift	3.35384	0.19	Y	O15	Open	3.32581	0.28	–
D11	Drift	3.33369	0.19	Y	O16	Open	3.32580	0.13	–
D12	Drift	3.33013	0.19	Y	O17	Open	3.32581	0.13	–
D13	Drift	3.33185	0.19	Y	O18	Open	3.32797	0.28	Y
D14	Drift	3.32462	0.19	–	O19	Open	3.07239	1.03	Y
D15	Drift	3.32581	0.19	–	O20	Open	3.32580	1.03	–
D16	Drift	3.32581	0.19	–	O21	Open	2.82628	1.03	Y
D17	Drift	3.32541	0.19	–	O22	Open	3.43213	2.06	Y
D18	Drift	3.32580	24.60	–	O23	Open	3.32580	4.11	–
D19	Drift	3.32580	3.53	–	O24	Open	0.67743	1.03	Y
D20	Drift	3.32580	24.60	–	O25	Open	0.01429	0.76	Y
D21	Drift	3.32580	3.53	–	O26	Open	2.04097	1.03	Y
D22	Drift	3.32580	0.09	–	S01	Short	0.04365	1.28	Y
D23	Drift	3.32580	0.09	–	S02	Short	0.00000	1.28	Y
D24	Drift	3.32580	0.09	–	S03	Short	3.45330	1.28	Y
D25	Drift	3.32580	0.18	–	S04	Short	2.16474	1.28	Y
D26	Drift	3.32580	0.36	–	S05	Short	2.16474	1.28	Y
D27	Drift	3.32580	0.09	–	S06	Short	0.76490	1.28	Y
D28	Drift	3.32580	0.09	–	S07	Short	0.58847	1.28	Y
D29	Drift	3.32580	0.09	–	S08	Short	3.46894	1.28	Y
D30	Drift	3.32580	0.09	–	S09	Short	3.40179	1.28	Y
D31	Drift	3.32580	0.07	–	S10	Short	2.73731	1.28	Y
D32	Drift	3.32580	0.07	–	S11	Short	2.91376	1.28	Y
D33	Drift	3.32580	0.07	–	S12	Short	2.73735	1.28	Y
D34	Drift	3.32580	0.09	–	S13	Short	0.58857	1.28	Y
O01	Open	5.26021	0.28	Y	S14	Short	3.42344	1.28	Y
O02	Open	2.81656	0.28	Y	S15	Short	3.42364	1.28	Y
O03	Open	1.97471	0.28	Y	S16	Short	5.57252	1.28	Y
O04	Open	3.34965	0.28	Y	S17	Short	5.57256	0.60	Y
O05	Open	3.43183	0.28	Y	S18	Short	2.16474	1.28	Y

## Appendix B. FMEA and Threshold-Selection Validation

### Appendix B.1. FMEA Fault-Occurrence Weights

Table A3 lists the FMEA failure rates used to build the occurrence-weighted faulty distribution ${\hat{f}}_{1}\left(s\right)$; each of the R=78 valid modes is normalized by the total $\sum \lambda_{r}=3.28\times 10^{-8}$. The component-to-FMEA mapping follows the circuit BOM; the expanded mode-level mapping ships with the released artifacts. The two power transistors dominate the occurrence distribution (56.3% combined); because their modes are amplitude-indistinguishable from healthy operation, the occurrence-weighted coverage (34.0%) is well below the per-case coverage (57.7%).

**Table A3:** FMEA failure rates and normalized occurrence weights for the 78 valid fault-injection modes, grouped by component category. Identical-component groups are counted by distinct signal behavior, not instance count: the four 324Ω resistors (identical responses) give 2 modes; the four 806Ω resistors give 8. Rate sums reflect physical instances; normalized weights sum to one.

Component	Code	Modes	Rate Sum	Weight	Fault Types
Transistor (4 modes, 56.3%)					
Si NPN transistor	PZT2222A	2	$9.22\times 10^{-9}$	0.281	drift
Si PNP transistor	PZT2907A	2	$9.22\times 10^{-9}$	0.281	drift
Capacitor (53 modes, 30.2%)					
Capacitor 10 nF (×15)	C1–C17	41	$8.26\times 10^{-9}$	0.252	drift, open, short
Capacitor 15 nF	C2	5	$7.56\times 10^{-10}$	0.023	drift, open, short
Capacitor 100 nF	C4	3	$5.73\times 10^{-10}$	0.017	drift, open, short
Capacitor 6.8 nF	C3	4	$3.14\times 10^{-10}$	0.010	drift, open, short
Resistor (21 modes, 13.5%)					
Resistor 806 Ω (×4)	R9,10,12,27	8	$1.47\times 10^{-9}$	0.045	drift, open
Resistor 324 Ω (×4)	R18,19,23,24	2	$1.47\times 10^{-9}$	0.045	drift, open
Resistor 50 kΩ (×2)	R15,25	2	$7.34\times 10^{-10}$	0.022	drift, open
Resistor 7.5 kΩ	R11	2	$3.67\times 10^{-10}$	0.011	drift, open
Resistor 3.01 kΩ	R3	2	$2.71\times 10^{-10}$	0.008	drift, open
Resistor 5.23 kΩ	R1	1	$2.97\times 10^{-11}$	0.001	drift
Resistor 11.5 kΩ	R20	1	$2.97\times 10^{-11}$	0.001	drift
Resistor 24.3 kΩ	R21	1	$2.97\times 10^{-11}$	0.001	drift
Resistor 1.58 kΩ	R2	1	$2.19\times 10^{-11}$	0.001	drift
Resistor 200 kΩ	R4	1	$2.19\times 10^{-11}$	0.001	drift
Total		78	$3.28\times 10^{-8}$	1.000	

### Appendix B.2. Threshold-Policy Comparison

This appendix compares three threshold-selection policies at the 70dB design SNR. The counter parameters are fixed to (X,Y,H)=(3, 2, 47), so that the comparison isolates the effect of the threshold-selection rule.

The first policy maximizes the separation $\Delta_{p}=p_{1}-p_{0}$. Although this policy improves the nominal separation between healthy and faulty samples, it places the threshold band inside the healthy distribution and produces a large healthy violation probability $p_{0}$. Consequently, the resulting false-confirmation probability exceeds the target.

The second policy directly imposes the finite-horizon constraint $P_{FA}\leq \alpha$. It produces a feasible threshold band, but its result is sensitive to the estimated distribution tails and to the selected numerical constraint.

The third policy contains the measured healthy operating envelope with a guard band. This rule keeps the empirical healthy violation probability near zero and provides a more robust engineering margin against measurement noise and temporal variability. It is therefore selected for the proposed monitor. The comparison is summarized in Table A4.

**Table A4:** Comparison of threshold-selection policies at the 70dB design SNR, with (X,Y,H)=(3, 2, 47).

Threshold Policy	*L*	*U*	$p_{0}$	$P_{\mathrm{FA}}\left(T_{h}\right)$
Maximize $p_{1}-p_{0}$	3.32505	3.32682	0.40	$7.2\times 10^{-1}$
Constrained $P_{FA}\leq \alpha$	3.32438	3.32750	0.14	$9.9\times 10^{-10}$
Healthy envelope + guard band	3.32437	3.32750	0.14	$9.4\times 10^{-10}$

### Appendix B.3. KDE Subsampling Sensitivity

The Simulink healthy record is subsampled to 50,000 points for KDE estimation. Table A5 confirms that the selected containment band and the healthy violation rate $p_{0}$ are insensitive to the subsample size: all sizes yield $p_{0}\approx 0$ and only sub-$10^{-4}$ changes in $L^{★},U^{★}$. The band reported here is recomputed directly from each subsample for this sensitivity check, so the 50k value (3.32426,3.32755) differs from the value used in the main text $\left(L^{★},U^{★}\right)=\left(3.32437,3.32750\right)$ only at the $10^{-4}$ level, within this insensitivity range.

**Table A5:** KDE subsampling sensitivity for the Simulink healthy record. The containment band $L^{★}=s_{\min}^{\left(0\right)}-3{\hat{\sigma }}_{0}$, $U^{★}=s_{\max}^{\left(0\right)}+3{\hat{\sigma }}_{0}$ and the healthy violation rate $p_{0}$ are recomputed at each subsample size; all sizes yield $p_{0}\approx 0$ and only small changes in the selected band.

Subsample Size	$L^{★}$	$U^{★}$	$p_{0}$	$|\Delta L^{★}|,\;|\Delta U^{★}|$ vs. 50 k
50 k	3.32426	3.32755	≈0	reference
100 k	3.32430	3.32763	≈0	$4.1\times 10^{-5}$, $8.0\times 10^{-5}$
500 k	3.32413	3.32766	≈0	$1.3\times 10^{-4}$, $1.1\times 10^{-4}$
Full ($8.06\times 10^{6}$)	3.32399	3.32774	≈0	$2.7\times 10^{-4}$, $1.9\times 10^{-4}$

### Appendix B.4. Empirical False-Confirmation Check on Collected Healthy Data

The optimized monitor $\Theta^{★}$ is applied to the collected healthy LVDT signal in non-overlapping windows of length $T_{h}=500$. Table A6 reports the full-record result and a held-out check (band re-estimated on the first half, evaluated on the unseen second half); neither produces a false confirmation. Transfer across flights, units, and operating temperature remains future work.

**Table A6:** Empirical false-confirmation check on collected healthy LVDT measurements.

Data Segment	Windows ($T_{h}=500$)	Trips	Empirical False-Confirmation Rate
Full healthy record	1116	0	0 (95% upper bound $2.68\times 10^{-3}$)
Held-out half	558	0	0 (95% upper bound $5.4\times 10^{-3}$)

## Appendix C. Numerical Verification and Search Efficiency

### Appendix C.1. Calibration-Model Order Verification

The measured healthy record contains 558,001 samples. For calibration, the steady-state portions of the measured and clean simulated signals were restricted to their common time interval. Then, the simulated signal was interpolated onto the measurement grid, and the two signals were temporally aligned. Affine, quadratic, and cubic calibration models were fitted to the resulting paired samples using the same ordinary least-squares criterion. For numerical stability, the higher-order models were fitted with internal signal scaling, and their coefficients were subsequently converted to the original signal scale.

The resulting calibration models are listed below, with all signal values expressed in volts.

- Affine model:
  $$s_{k}^{\mathrm{cal}}=0.907375\;s_{k}^{\mathrm{sim}}+0.665201.$$
- Quadratic model:
  $$s_{k}^{\mathrm{cal}}=A{\left(s_{k}^{\mathrm{sim}}\right)}^{2}+B\;s_{k}^{\mathrm{sim}}+C,$$
  where
  A=−908.88,B=5331.28,C=−7814.71.
- Cubic model:
  $$s_{k}^{\mathrm{cal}}=A{\left(s_{k}^{\mathrm{sim}}\right)}^{3}+B{\left(s_{k}^{\mathrm{sim}}\right)}^{2}+C\;s_{k}^{\mathrm{sim}}+D,$$
  where
  A=13086858.94,B=−115128416.46,
  C=337604624.72,D=−329999274.65.

The selected affine model achieved a calibration SDR of 85.48dB. The residual errors of the three model orders are compared in Table A7.

**Table A7:** Calibration-model order comparison using the same paired healthy samples obtained after common-interval selection, interpolation, and temporal alignment.

Model	Residual RMS (V)	Reduction vs. Affine
Affine	$1.770142\times 10^{-4}$	Reference
Quadratic	$1.770064\times 10^{-4}$	0.0044%
Cubic	$1.770042\times 10^{-4}$	0.0057%

The quadratic and cubic models reduced the residual RMS relative to the affine model by only 0.0044% and 0.0057%, respectively. These improvements are too small to justify the additional model parameters. Therefore, the affine gain-bias model was selected for mapping the Simulink outputs to the measured-data scale.

### Appendix C.2. Numerical Verification of DTMC Reliability Evaluation

Before being used for synthesis, the finite-horizon DTMC evaluation is checked against Monte Carlo simulation. For representative configurations whose targets are statistically resolvable with $N_{\mathrm{MC}}=10^{6}$ Bernoulli-input trials at the same $p_{0}\left(L,U\right)$ and $p_{1}\left(L,U\right)$, $P_{FA}$ and $P_{MD}$ (from Equation (33)) are compared with the empirical estimates, together with an ADD check for $\Theta^{★}$ (Table A8). The agreement verifies the numerical implementation under the assumed Bernoulli model; it does not validate the i.i.d. assumption for temporally correlated measured violations, discussed in Section 4.4.

**Table A8:** Numerical verification of DTMC reliability evaluation against Monte Carlo simulation.

Configuration	Metric	DTMC	Monte Carlo	Status
$\Theta_{FA}$	$P_{FA}$	$6.55\times 10^{-1}$	$6.55\times 10^{-1}$, CI [0.654,0.656]	pass
$\Theta_{MD}$	$P_{MD}$	$2.95\times 10^{-1}$	$2.94\times 10^{-1}$, CI [0.293,0.295]	pass
$\Theta^{★}$	ADD	33.30	33.30, CI [33.24,33.35]	pass

### Appendix C.3. Efficiency of Reachable-State Pruning

The offline search uses X∈{1,…,50}, Y∈{1,…,5}, and H∈{1,…,100}, with H>max(X,Y), resulting in 18,605 valid configurations. Reachability pruning identifies 5925 configurations (31.8%) containing unreachable states. The mean full-state/reachable-state ratio is 1.55×, corresponding to a 35.5% reduction in propagated states; the maximum ratio is 4.81×, corresponding to a 79.2% reduction. Both implementations produce identical $P_{FA}$, $P_{MD}$, and ADD values. Table A9 summarizes the search grid and the resulting reachable-state pruning statistics.

**Table A9:** Search grid and reachable-state pruning results.

Quantity	Value
*X*	{1,2,…,50}
*Y*	{1,2,…,5}
*H*	{1,2,…,100}
Constraint	H>max(X,Y)
Configurations	18,605
Unreachable-state cases	5925 (31.8%)
Mean ratio	1.55×
Maximum ratio	4.81×
(X,Y,H)=(3, 2, 47)	48/48 states reachable

## References

[1] Izadi I., Shah S.L., Shook D.S., Chen T. (2009). An introduction to alarm analysis and design. IFAC Proc. Vol..

[2] (2016). Management of Alarm Systems for the Process Industries.

[3] EEMUA (2013). Alarm Systems: A Guide to Design, Management and Procurement.

[4] Wang J., Hu W., Chen T. (2024). Intelligent Industrial Alarm Systems: Advanced Analysis and Design Methods.

[5] Adnan N.A., Izadi I., Chen T. (2011). On expected detection delays for alarm systems with deadbands and delay-timers. J. Process Control.

[6] Xu J., Wang J., Izadi I., Chen T. (2012). Performance Assessment and Design for Univariate Alarm Systems Based on FAR, MAR, and AAD. IEEE Trans. Autom. Sci. Eng..

[7] Afzal M.S., Chen T., Bandehkhoda A., Izadi I. (2018). Analysis and Design of Time-Deadbands for Univariate Alarm Systems. Control Eng. Pract..

[8] Adnan N.A., Cheng Y., Izadi I., Chen T. (2013). Study of generalized delay-timers in alarm configuration. J. Process Control.

[9] Zang H., Yang F., Huang D. (2015). Design and analysis of improved alarm delay-timers. IFAC-PapersOnLine.

[10] Tulsyan A., Alrowaie F., Gopaluni B. (2018). Design and assessment of delay timer alarm systems for nonlinear chemical processes. AIChE J..

[11] Tulsyan A., Gopaluni R.B. (2019). Univariate model-based deadband alarm design for nonlinear processes. Ind. Eng. Chem. Res..

[12] Zhang Z., Wang J., Qi Y. (2024). Selection of alarm deadbands and delay timers with their connections based on risk indicators for removing nuisance alarms. Control Eng. Pract..

[13] Zhou J., Shang J., Chen T. (2025). Performance Analysis for Stochastic Anomaly Detectors with On/Off-Delay Timers. Automatica.

[14] Raei R., Izadi I., Kamali M. (2023). Performance Analysis of Up/Down Counters in Alarm Design. Process Saf. Environ. Prot..

[15] Rahimi Y., Mohan Rao H.R., Chen T. Optimizing Alarm Design: A Comparison of Delay-Timers, Counters, and Time-Deadbands. Proceedings of the 2024 IEEE 3rd Industrial Electronics Society Annual On-Line Conference (ONCON).

[16] Aslansefat K., Gogani M.B., Kabir S., Shoorehdeli M.A., Yari M. (2020). Performance Evaluation and Design for Variable Threshold Alarm Systems through Semi-Markov Process. ISA Trans..

[17] Gyasi P., Wang J. (2023). Design of Alarm Thresholds and Delay Timers for Non-IID Process Variables Based on Alarm Durations. Process Saf. Environ. Prot..

[18] Asaadi M., Izadi I., Hassanzadeh A., Yang F. (2022). Assessment of Alarm Systems for Mixture Processes and Intermittent Faults. J. Process Control.

[19] Page E.S. (1954). Continuous inspection schemes. Biometrika.

[20] Wald A. (1947). Sequential Analysis.

[21] Lorden G. (1971). Procedures for reacting to a change in distribution. Ann. Math. Stat..

[22] Moustakides G.V. (1986). Optimal stopping times for detecting changes in distributions. Ann. Stat..

[23] Fu J.C., Shmueli G., Chang Y.M. (2003). A unified Markov chain approach for computing the run length distribution in control charts with simple or compound rules. Stat. Probab. Lett..

[24] Ozdemir A.A., Seiler P., Balas G.J. (2011). Wind turbine fault detection using counter-based residual thresholding. IFAC Proc. Vol..

[25] Ford R.M., Weissbach R.S., Loker D.R. (2001). A novel DSP-based LVDT signal conditioner. IEEE Trans. Instrum. Meas..

[26] Flammini A., Marioli D., Sisinni E., Taroni A. (2007). Least Mean Square Method for LVDT Signal Processing. IEEE Trans. Instrum. Meas..

[27] Goupil P. (2011). AIRBUS state of the art and practices on FDI and FTC in flight control system. Control Eng. Pract..

[28] Marzat J., Piet-Lahanier H., Damongeot F., Walter E. (2012). Model-based fault diagnosis for aerospace systems: A survey. Proc. Inst. Mech. Eng. G J. Aerospace Eng..

[29] Fu S., Avdelidis N.P. (2023). Prognostic and Health Management of Critical Aircraft Systems and Components: An Overview. Sensors.

[30] Zong G., Yang D., Lam J., Song X. (2022). Fault-Tolerant Control of Switched LPV Systems: A Bumpless Transfer Approach. IEEE/ASME Trans. Mechatron..

[31] Zong G., Xie H., Yang D., Zhao X., Yi Y. (2024). Adaptive Fuzzy Tracking Control for Switched Nonlinear Systems Under FDI Attacks and Input Saturation: A Flexible Transient Performance Approach. IEEE Trans. Cybern..

[32] Zhang L., Dong J. (2026). Guaranteed State and Fault Interval Estimation for Local Nonlinear Takagi–Sugeno Fuzzy Systems Based on Zonotopic Analysis. IEEE Trans. Fuzzy Syst..

[33] Parzen E. (1962). On estimation of a probability density function and mode. Ann. Math. Stat..

[34] Silverman B.W. (1986). Density Estimation for Statistics and Data Analysis.

[35] McLachlan G.J., Peel D. (2000). Finite Mixture Models.

[36] (2023). Guidelines for Conducting the Safety Assessment Process on Civil Aircraft, Systems, and Equipment.

[37] (2024). System Design and Analysis.

